# Annotation of the Protein Coding Regions of the Equine Genome

**DOI:** 10.1371/journal.pone.0124375

**Published:** 2015-06-24

**Authors:** Matthew S. Hestand, Theodore S. Kalbfleisch, Stephen J. Coleman, Zheng Zeng, Jinze Liu, Ludovic Orlando, James N. MacLeod

**Affiliations:** 1 Maxwell H. Gluck Equine Research Center, Department of Veterinary Science, University of Kentucky, Lexington, Kentucky, United States of America; 2 Biochemistry and Molecular Biology Department, School of Medicine, University of Louisville, Louisville, Kentucky, United States of America; 3 Department of Computer Science, University of Kentucky, Lexington, Kentucky, United States of America; 4 Centre for GeoGenetics, Natural History Museum of Denmark, University of Copenhagen, Copenhagen, Denmark; Hellas, GREECE

## Abstract

Current gene annotation of the horse genome is largely derived from *in silico* predictions and cross-species alignments. Only a small number of genes are annotated based on equine EST and mRNA sequences. To expand the number of equine genes annotated from equine experimental evidence, we sequenced mRNA from a pool of forty-three different tissues. From these, we derived the structures of 68,594 transcripts. In addition, we identified 301,829 positions with SNPs or small indels within these transcripts relative to EquCab2. Interestingly, 780 variants extend the open reading frame of the transcript and appear to be small errors in the equine reference genome, since they are also identified as homozygous variants by genomic DNA resequencing of the reference horse. Taken together, we provide a resource of equine mRNA structures and protein coding variants that will enhance equine and cross-species transcriptional and genomic comparisons.

## Introduction

Complete sequencing of any genome, including the horse [1], is a major accomplishment. In order to make full use of the genome sequence, the structural and functional features it encodes need to be identified and described, requiring layers of reliable annotation. One of the most fundamental types of annotation is the structure and location of genes, based on knowledge of the encoded transcripts. When only a genome sequence and limited experimental data are available, *in silico* predictions are a useful tool, but these tools are not perfect. Alternatively, alignment of transcripts from other species is also frequently being used to predict structures, but interspecies variation still results in considerable errors. Experimental approaches traditionally include the sequencing and alignment of mRNAs or ESTs, or more recently using next-generation sequencing of RNA (RNA-seq [2]). A second type of annotation is genomic sequence variations, including single nucleotide polymorphisms (SNPs) and larger insertions or deletions (indels) compared to the reference genome. The first horse genome sequenced was a Thoroughbred [1], but recently several other breeds have been sequenced, including the Arabian, Duelmener, Hanoverian, Icelandic, Norwegian Fjord, Przewalski, Quarter Horse, Sorraia, and Standardbred [3–5]. This revealed millions of SNP variants and indels that had previously been unannotated in the reference genome. Messenger RNA sequencing data collected from multiple horses to study transcript structures can also be used to identify variants in the protein coding regions of the genome.

The level of annotation in the equine genome is similar to many other species. A reference genome has been assembled, yet very limited expression data exist to describe gene structure. Accordingly, a majority of the protein-coding gene annotations are currently based on *in silico* predictions and cross-species comparisons. Messenger RNA from eight equine tissue samples was sequenced using the Illumina platform and the resulting data used to refine structural annotations at 11,356 protein-coding loci [6]. This work represented the first transcriptome-level effort with equine-specific experimental data to improve the structural annotation of protein-coding genes in the horse. Due to the limited sample set, not all genes were represented and those that were transcribed may express additional mRNA variants through alternative splicing and other mechanisms in different cell types. In order to get a more comprehensive view of gene and transcript structure and continue efforts to improve annotation of the equine genome, a larger panel of tissues must be queried. Here we present the sequencing of mRNAs from 43 equine tissues. The analyses enabled the assembly of transcript structures and identification of protein-coding variants, some of which likely indicate small errors in the reference genome.

## Results

### Sequencing, alignment, and transcript construction

A pooled mRNA sample consisting of 43 different equine tissues was sequenced using the Illumina HiSeq platform, producing 142,596,524 single end 75bp reads and 2×139,181,480 paired-end 75bp reads. The mRNA pool was also sequenced on the 454 platform, producing 1,777,191 reads. All sequencing reads are available in the ArrayExpress database [7] (www.ebi.ac.uk/arrayexpress) under accession number E-MTAB-2879. After quality and linker sequence trimming, 345,268,823 Illumina reads aligned uniquely, and 15,100,254 reads aligned to multiple genomic locations.

Initial transcript reconstruction of the Illumina reads resulted in 144,968 transcripts. This number was reduced to 144,109 transcripts using 454 reads (post-filtering mean length 320bp, median length 308bp) and non-canonical junctions (i.e. non GT-AG splice junctions) to identify introns (i.e. exon-exon links) missed in the original reconstruction. Of these putative transcripts, 110,451 were single exon transcripts and 7,116 transcripts were composed of two exons. Those that did not resemble annotated Ensembl [8] genes or any of the 454 reads, as determined by BLAST [9], were excluded from further analyses (72,809 excluded one exon and 2,706 excluded two exon transcripts). The final set of 68,594 transcripts was composed of 37,642 single exon transcripts, 4,410 two-exon transcripts, and 26,542 transcripts with three or more exons. A gtf format file of the transcript structures is available at http://dx.doi.org/10.13013/J6057CVW.

### Transcript evaluation

**Fig 1:**
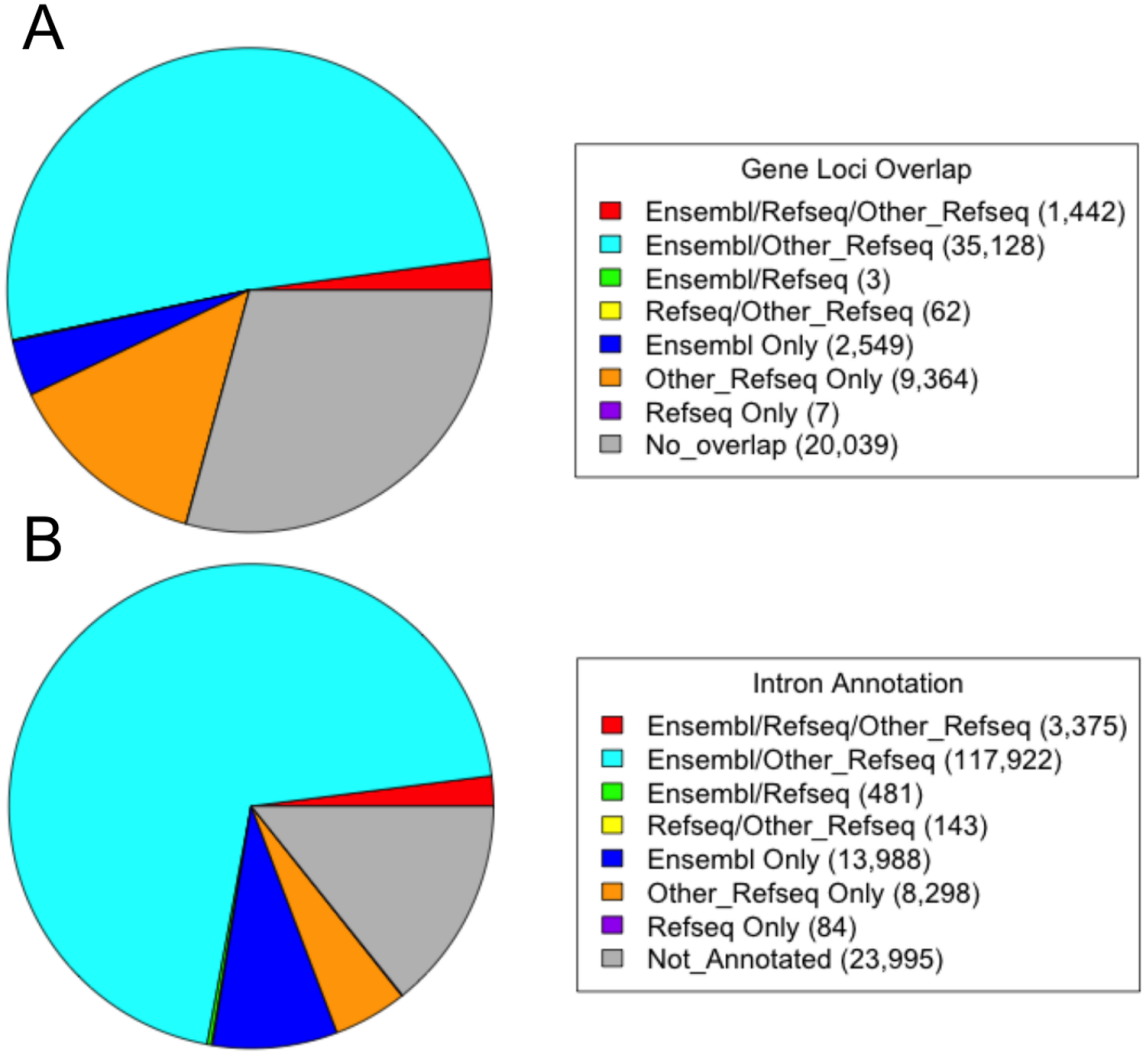
Overlap of RNA-seq defined transcript structures with annotated gene loci (A) and annotated introns (B). For gene loci overlap, numbers indicate transcripts overlapping Ensembl, Refseq, or other Refseq gene loci only, or the number overlapping multiple annotation sources. For intron overlap, numbers indicate exact identity between the splice donor and acceptor sites in the RNA-seq data with genomic intron positions in one source only or the number found identical in multiple annotation sources.

The reconstructed transcripts were evaluated by comparison to available gene annotations. We found that 71% (48,555 out of 68,594) of transcripts overlapped previous annotated gene loci (Fig 1A). More specifically, 39,122 (57%) overlap an Ensembl equine gene loci, 1,514 (2%) overlap a Refseq equine gene loci, and 45,996 (67%) overlap a gene loci defined from a cross-species Refseq gene. A majority of the transcripts from previously unannotated loci (91%, 18,201 of 20,039) are composed of a single exon. Forty percent (7,995 of 20,039) of transcripts that did not overlap with annotated loci are also located within the unassigned genomic DNA contigs of chrUn. As expected, most (7,582) of these were composed of only a single exon.

The reconstructed transcript structures were also evaluated by comparing the concordance of specific splice donor and splice acceptor pairs with previously annotated introns. We believe this is an appropriate method for evaluating the derived transcript structures given the large variability in 5′ starts of first exons and 3′ ends of last exons. Of the 168,286 introns identified in our transcript structures, 135,766 (81%) were annotated by Ensembl, 4,083 (2%) were annotated in RefSeq, and 129,738 (77%) were annotated by cross-species RefSeq alignments (Fig 1B).

**Fig 2:**
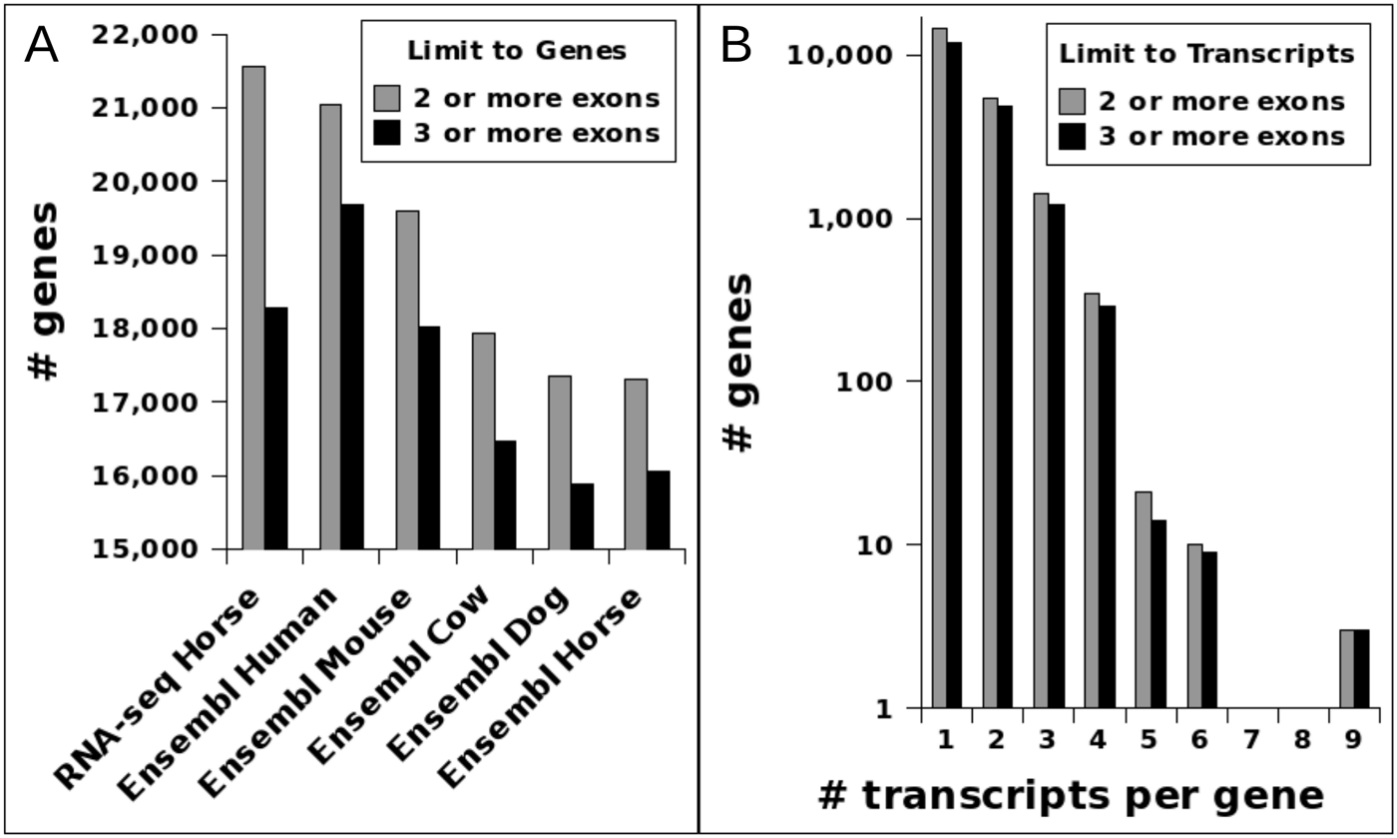
The number of gene loci annotated in multiple organisms (A) and the number of equine RNA-seq based transcripts per gene loci (B). These have been selected for transcripts or gene loci with two or more exons or three or more exons. Gene numbers for non-horse RNA-seq data are from Ensembl 73 Biomart queries.

**Fig 3:**
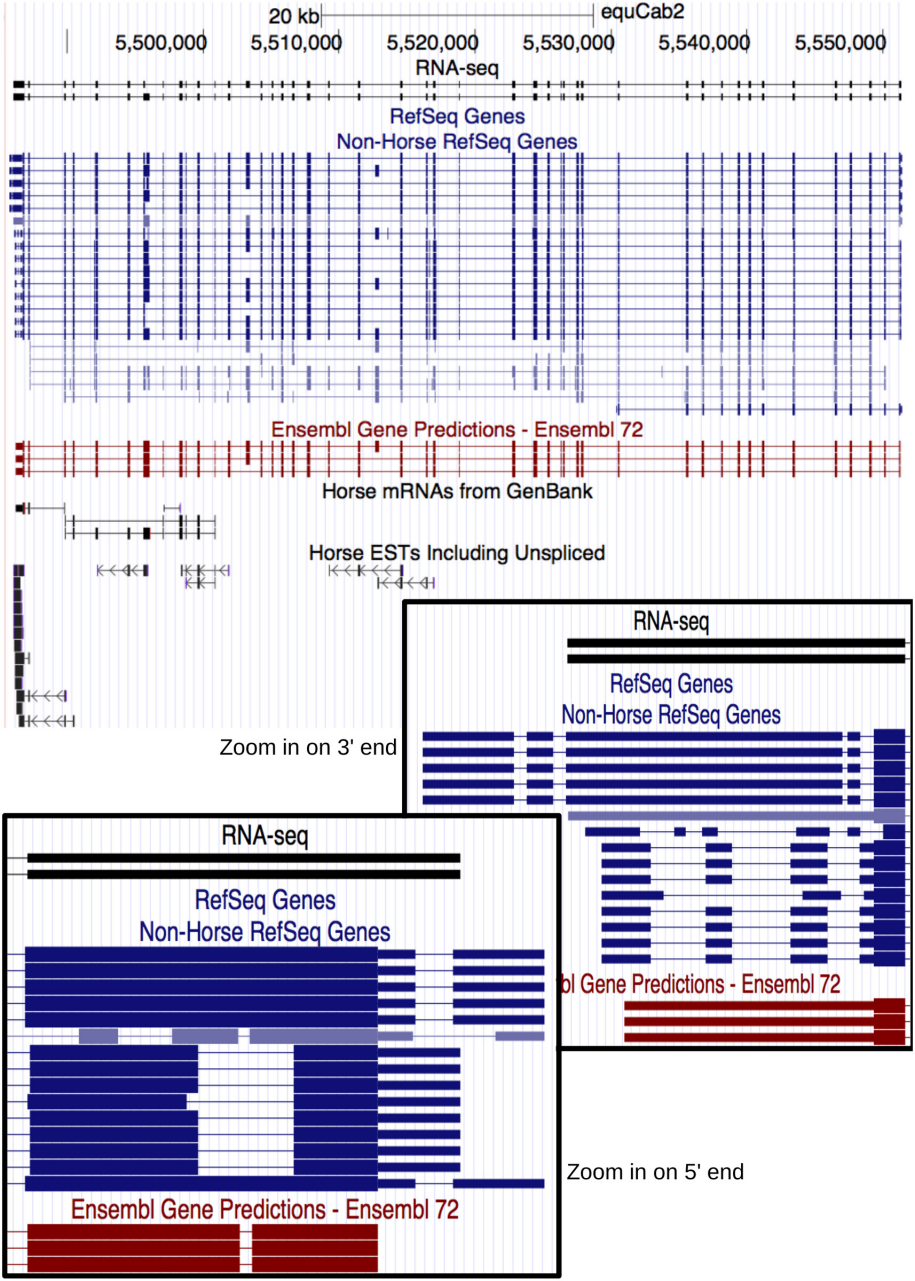
UCSC Screenshot of FN1 RNA-seq defined transcripts. This gene loci, previously only supported by limited equine-specific transcriptional data (ESTs and mRNA fragments), now shows experimental evidence for two transcripts. It also refines structures predicted by cross-species alignments and shows refinements over 5′ and 3′ ends of Ensembl predicted transcripts.

The number of gene loci with an identified transcript of at least 2 exons (2 or more exons) totaled 21,569. The subset of these gene loci expressing a transcript of at least 3 exons (3 or more exons) totaled 18,284. These numbers are similar to the Ensembl 73 annotation of human and mouse, while exceeding those of cow, dog, and previous horse annotations (Fig 2A). Approximately 66% of gene loci (14,409 gene loci with at least 2 exons, or 11,911 gene loci with at least 3 exons) contained only a single transcript. The remaining gene loci showed alternative splicing, with a maximum of 9 transcripts identified from a single gene locus (Fig 2B). This included full length transcripts from genes composed of many exons, such as the FN1 gene loci (Fig 3).

### Variant detection

Nucleotide sequence variants between the transcriptional data and the equine reference genome sequence were detected and analyzed relative to RNA-seq derived gene structures. These variants included SNPs, insertions, and deletions. Since the individual samples in the sequenced RNA pool were not barcoded, it is not possible to identify if a variant is heterozygous or represented by two different horses. We can therefore classify homozygous variants unambiguously, but at positions where two alleles are detected, we can only state that the second allele is present in horses. It cannot be determined from the current dataset whether the second allele is tolerated as a homozygote. A total of 126,392 homozygous variant positions and 175,437 mixed allele variant positions were identified in the RNA-seq reads. Some variant positions identified more than one variant, with a total of thirteen percent of variants (39,860 of 302,585) previously annotated in Ensembl. Forty-seven percent (32,379) of the RNA-seq transcripts contained at least one variant. When these variants were compared to the reconstructed transcripts, 528 had a longer open-reading frame (ORF) when incorporating one of the SNPs, 536 had a longer ORF when incorporating an insertion, and 585 had a longer ORF when incorporating a deletion. In addition, one transcript had both a SNP and deletion that increased the length of its ORF.

Genomic resequencing of the horse Twilight [10], from which the reference was created, detected 12,866 homozygous variant positions, 91,671 bi-allelic variant positions, and 112 other variant positions within the exons identified by the RNA-seq data. Twenty-nine percent of the variants identified within these loci (30,771 of 105,717) had previously been annotated in Ensembl. A more comprehensive evaluation was performed against variants in the five breeds identified in Orlando *et al*. 2013 [3] (Table 1). Fifty seven percent of RNA-seq variants were found in at least one horse from the Orlando *et al*. 2013 dataset. When including the Twilight resequencing data, the number of RNA-seq variants found in at least one other horse increased to 66%. This suggests that the list of SNP variants derived from our RNA-seq sequence data and those derived from independent genomic surveys are consistent and represent genuine sequence variation within horse breeds. Both transcriptomic and genomic vcf files are available at http://dx.doi.org/10.13013/J6057CVW.

**Table 1:** Overlap of Variants with Additional Horse Datasets.

not inc. Twilight	1	2	3	4	5		not validated
SNPs	43,774	37,141	34,414,	31,445	19,657		126,439
Insertions	585	541	481	526	317		2,378
Deletions	790	620	575	537	380		1,985
inc. Twilight	1	2	3	4	5	6	not validated
SNPs	44,116	36,545	32,541	30,553	25,102	11,951	112,062
Insertions	1,226	561	504	470	481	286	1,300
Deletions	1,394	679	563	524	505	358	864

The top rows indicate the number of variants discovered by RNA-seq also found in one, two, three, four, or all five of the breeds identified in Orlando *et al*. 2013 [3]. The bottom rows indicate the number found in one to six of the breeds in Orlando *et al*. 2013 and the resequencing data of the reference horse, Twilight, in Rebolledo-Mendez *et al*. 2015 [10].

**Fig 4:**
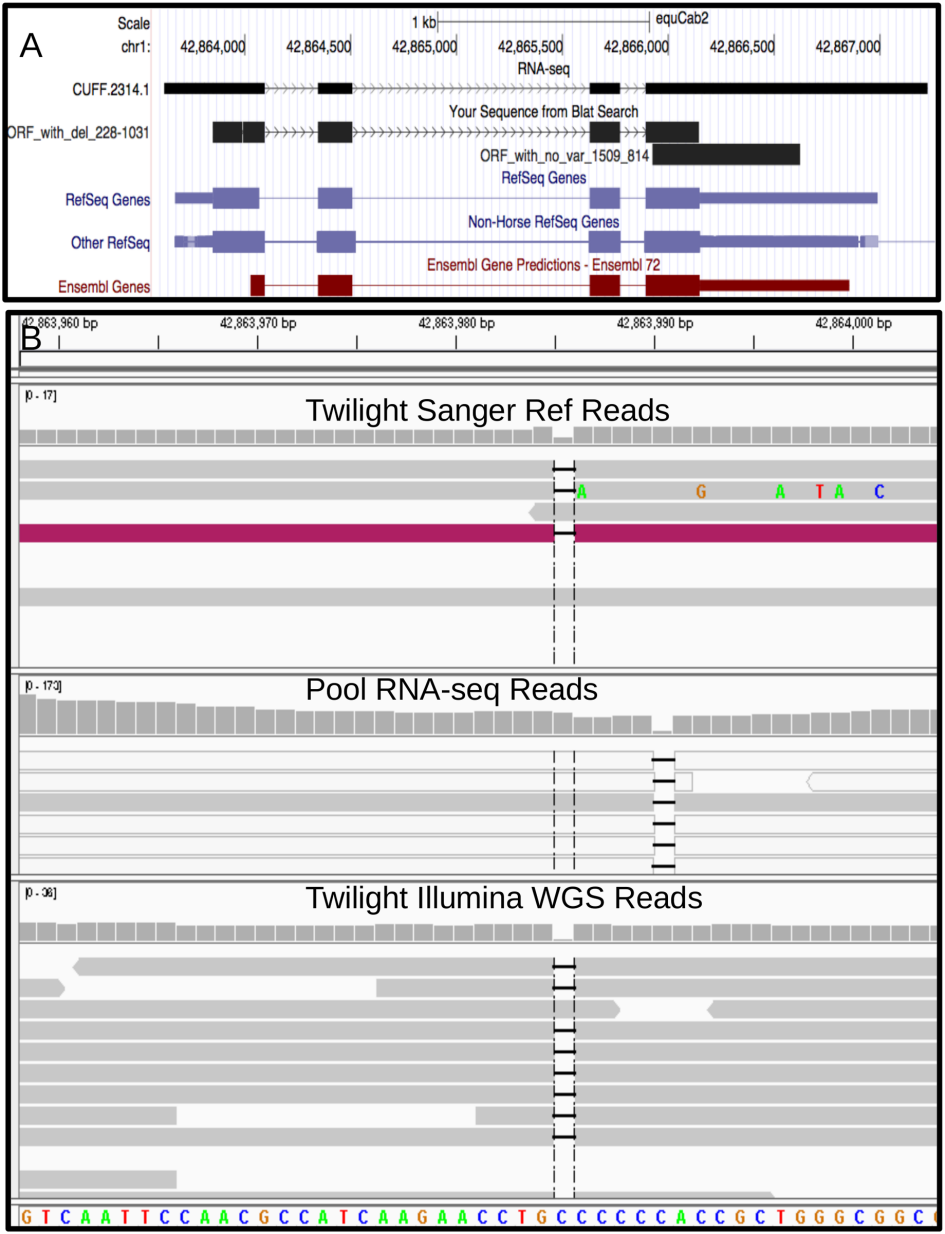
An example of a deletion (chr1:42863989, CC > C) that extends the ORF in the gene DKK1. In UCSC (A), the full constructed transcript is shown as the top track, followed by a BLAT track showing the ORF with (ORF_with_del_228-1031) and without (ORF_with_no_var_1509_814) the deletion. The transcript structure can be seen to resemble the known annotations, and the ORF is similar to annotation only when including the deletion. In IGV [11] (B) it can be seen that the deletion event is present in three out of five Sanger reads, as well as most Illumina genomic resequencing and RNA-seq reads. Note: though the position of the deletion is the first C in a stretch of C’s within the genomic reads and the last C in the RNA-seq reads, this gives the same result.

Twenty percent of the ORF extending SNPs found by RNA-seq were confirmed in the Twilight genomic data (Table 2). Additionally, 66% of the ORF extending insertions and 71% of the ORF extending deletions found in the RNA-seq data were supported by genomic analyses (Table 2). Of the 780 variants that were identified as homozygous in the reference animal and extended the open-reading frame of the transcript they reside in (S1 Dataset), most were not SNPs, but small indels. One such example is a 1bp deletion (chr1:42863989 CC > C) within the DKK1 gene loci, supported by 67 of 77 RNA-seq reads and all 23 genomic reads (vcf AD fields) (Fig 4).

**Table 2:** Number of ORF Extending Variants in RNA-seq Data.

	SNPs	Insertions	Deletions
Reference Homozygous Genomic Variant	20	333	427
Reference Heterozygous Genomic Variant	88	33	13
Not Supported by Genomic Resequencing	444	186	178

Indicated is the number of variants discovered by RNA-seq that extend ORFs of an RNA-seq defined transcript, broken down by type of validation from reference horse genomic resequencing.

## Discussion

Next-generation sequencing has quickly become established as a standard tool for refining genome sequence assemblies and annotation. The technology is particularly valuable for smaller research communities, like those studying the horse, which would otherwise lack the necessary species-specific expression data required for annotation. The small number of mRNAs/ESTs sequenced using earlier technologies explains the small level of overlap with equine Refseq gene loci and introns (Fig 1). In the current study, RNA-seq was applied to a pooled sample composed of RNA isolated from 43 equine tissue samples to analyze equine transcripts and further advance efforts to annotate the equine genome.

Preliminary analysis of the sequencing results generated 144,968 assembled transcripts. The total number considered in our analysis was quickly reduced by filtering out single and two exon transcripts that did not show similarity to an Ensembl gene annotation or any of the independently generated 454 sequencing data. The filtered transcripts could be indicative of mapping and assembly errors, or may represent non-coding RNAs. The result of the filtering was a final set of 68,594 putative mRNA transcripts. Overall, 71% (48,555 of 68,594) of the transcripts from this study overlap a known gene locus (Fig 1A), with 21,569 gene loci having transcripts with at least 2 exons and 18,284 gene loci having transcripts with at least 3 exons. Since these numbers are similar to the annotations of human and mouse, while exceeding those of the less studied organisms (cow, dog, and previous horse annotations, Fig 2A), we believe this pool of 43 different tissues enabled us to reach a near complete coverage of gene loci with broad patterns of expression. However, the observation of a larger ratio of equine genes with at least 2 exons compared to 3 exons relative to human and mouse gene structures (Fig 2A) suggests that additional data, and especially longer reads, will be necessary to complete gene annotation in horses.

Forty percent of transcripts that did not overlap with annotated loci were located within the unassigned genomic DNA contigs of chrUn and were mostly composed of a single exon. It is likely that these small genomic fragments contain only parts of genes, explaining the high frequency of their identification in the current study and why they were not previously annotated. The single exon transcripts that did not overlap with annotated loci and were on autosomes or chromosome X could represent non-coding RNAs (such as long non-coding RNAs or others) or more recently described small open reading frames (smORFs) [12, 13]. Still, we believe this to be a robust transcript assembly, with more than 85% (144,291 of 168,286) of introns identified in the RNA-seq data concordant with previous annotations (Fig 1B). We were also able to reconstruct full-length transcripts, including gene loci with alternatively spliced transcripts. One such example is FN1 (Fig 3), which encodes the large extracellular matrix protein fibronectin and had previously been supported by only partial equine specific data (mRNAs/ESTs). The FN1 gene expresses multiple mRNA variants through alternative splicing [14]. RNA-seq data from the pooled sample used in the current study detected highly expressed splice variants involving extra type III domain A (ED-A) and the variable (V) regions, but not more tissue-restricted transcripts such as the (V+C)- splice variant expressed by chondrocytes [15]. Since the Cufflinks algorithm tries to find the minimal number of transcripts that may explain the reads, tissue specific mRNAs diluted in the pool can be lost in the analysis. Therefore, additional studies that target individual tissues, cell types, developmental time points, and physiological/pathological conditions will greatly facilitate the structural characterization of mRNA transcripts expressed in highly restricted patterns.

In addition to improving the experimental evidence available for equine transcript structures, this RNA-seq data identified sequence variants at over 300,000 positions, approximately a third of which were also identified in genomic re-sequencing of the reference genome horse. Interestingly, only 13% and 29% of the transcriptomic and genomic variants, respectively, were annotated. The transcriptomic variants could be explained by the lack of other individual horses sequenced in the past, but to identify such a large number of novel variants from the reference animal was surprising. We believe many of the variants that are found in the RNA-seq reads and also homozygous in the reference individual are likely Sanger sequencing errors. One example, as seen in Fig 4, had only two of five Sanger reads supporting the reference, indicating an error in calling the bases from the original trace files. This deletion variant is also found as a homozygous variant in previously published Icelandic and Arabian horse genome sequences [3], When not including the deletion, the longest ORF is just a stretch within the 3′ most exon. However, when the transcript sequence is corrected by removing the additional cytosine the ORF is a near match to those of Refseq and Other Refseq. This deletion is within a stretch of C nucleotides. Errors in calling the number of nucleotides in stretches of homopolymers is a common problem for traditional Sanger sequencing, which was the method used with the reference genome EquCab2 [1]. Indeed, of the 806 ORF extending indels found in both RNA-seq and genomic data, 718 added or subtracted the same nucleotide as an adjacent nucleotide. Evidence for these being errors in the reference is also supported by the fact that 69% (306) of the deletions are found within 10bp of an annotated intron of the same size.

In conclusion, we present 68,594 equine transcript structures based on RNA-seq data generated from 43 horse tissue samples. This new layer of annotation, together with the identification of over 300k variant positions, will benefit the horse community and scientists using comparative genomic approaches across a diversity of mammalian species.

## Materials and Methods

### Ethics Statement

This study was carried out in accordance with experimental protocol 00814A2004 approved by the University of Kentucky’s Institutional Animal Care and Use Committee (PHS Assurance #A3336-01). Euthanasia was performed as recommended by the American Veterinary Medical Association using a commercial Sodium Pentobarbital euthanasia solution administered intravenously following the manufacturer’s guidelines.

### RNA isolation and sequencing

Total RNA was isolated from 43 equine tissue samples (S2 Dataset) collected from 11 individual horses using previously reported methods [15, 16]. All tissues were considered normal and careful efforts were in place to minimize any distortion of transcript levels caused by the collection procedure. Two micrograms of total RNA from each tissue sample were used to generate the final RNA pool. RNA quantity, purity, and integrity of both the individual samples and the resulting pool were determined with an Agilent 2100 Bioanalyzer using the Eukaryotic Total RNA nano series II analysis kit (S2 Dataset).

RNA was poly-A selected and used for an Illumina random priming directional library prep. However, downstream analyses (not shown) indicated that the strand selection was not effective and these data were subsequently not treated as strand specific. The library was sequenced on an Illumina HiSeq 2000 for 75bp reads. Four lanes were sequenced only on one end providing single end reads and 4 lanes were sequenced at both ends giving paired-end reads. Base calling and processing was performed using the Illumina pipeline version 1.7.

The RNA pool was also sequenced independently on a 454 Genome Sequencer FLX+ system as follows: messenger RNA was isolated from total RNA with the Oligotex kit (Qiagen, Valencia, CA) and converted to a primary cDNA library with adaptors compatible with the 454 system as previously published [17]. A second library was constructed with the ScriptSeq mRNA-Seq Library Preparation kit Roche Titanium-compatible (Epicentre, WI). Libraries were quantified using a Qubit fluorometer (Invitrogen, CA) and average fragment sizes were determined on an Agilent Bioanalyzer using a DNA 7500 chip. The libraries were diluted to 1×10^6^ molecules/*μ*l. Emulsion-based clonal amplification and sequencing was performed according to the manufacturer’s instructions (454 Life Sciences, Branford, CT). Signal processing and base calling were performed using the bundled 454 Data Analysis Software v2.6.

### Illumina read trimming and alignment of RNA-seq reads

Illumina reads were trimmed on the 5′ and 3′ ends to remove nucleotides with sequence N and/or quality score B using a custom Perl script. These reads were then trimmed for 3′ sequencing linkers using an updated and customized version of the GAPSS linker trimming script [18]. The reads were then aligned to NCBI build 2.2 of the equine genome (chromosomes 1–31, MT, Un, and X) using MapSplice 2.0 beta [19]. The following non-default settings were used: minimum entropy of 0, search for non-canonical in addition to canonical splice junctions, run across 12 threads, use a minimum read length of 35bp, insertion size of 3bp, and deletion size of 10bp. The SAM file was filtered for reads with more than 3 mismatches and for paired-end reads where both ends did not map within expected distances.

### Transcript reconstruction and evaluation

Transcripts were assembled from the final processed SAM file using Cufflinks v1.3.0 [20]. This was run using 12 processors and using the option to correct for multiple mappings (–multi-read-correct). We refined the Cufflinks transcripts by combining transcripts based on the 454 sequence reads as follows: As a first check, we identified 454 reads whose ends were in different Cufflinks transcripts. 454 sff data were converted to fastq files with sff extract v0.2.13 (http://bioinf.comav.upv.es/sff_extract/). Reads with a minimum length of 50nt were converted to 2×25bp paired-end reads, filtered for a minimum read quality of 28, and aligned with Bowtie v0.12.7 [21] (permitting up to 3 alignments per read and a maximum of 1 mismatch) to the Cufflinks transcripts. The 25bp paired-end segments from the 454 reads were considered potentially linking if they were in proper orientation and linked transcripts on the same genomic strand and the transcripts were located within 200,000bp of each other. After identifying reads whose ends linked two Cufflinks transcripts, we took the full length read sequences of these 454 reads and refined the transcript connections using BLAT v.34 [22] and filtered the connections for suggested introns within 10bp of an Ensembl, RefSeq, or other RefSeq intron (UCSC table browser [23–25] download August 2012). The results were manually evaluated relative to the Cufflinks transcripts to verify the connections.

As Cufflinks v1.3.0 did not utilize the non-canonical splice junctions provided by MapSplice, a custom Perl script was used to combine the Cufflinks transcripts which were connected by a non-canonical MapSplice splice junction.

BLAST was used to filter the large number of single and two exon transcripts generated by the Cufflinks analysis. Transcript sequences were aligned to a BLAST database comprised of the 454 reads and the protein coding cDNA sequences for horse, cow, dog, and human downloaded from Ensembl 67 Biomart [8]. A bit score of 400 was empirically determined to be an appropriate cut-off to retain as many multi-exon transcripts as possible while removing shorter transcripts (S1 Fig).

The RNA-seq based transcript structures were compared to previously available equine gene structure annotation. Annotated structures of whole genes from Ensembl, RefSeq, and Other RefSeq were downloaded in BED file format using the UCSC table browser (download April 2013). Overlap between the RNA-seq transcripts and annotated gene loci was evaluated with custom scripts to determine if the chr:start-stop position of the RNA-seq transcript overlapped the chr:start-stop position of an annotated gene. Similarly, the intron annotation was evaluated to determine if the chr:start-stop position and strand of a RNA-seq based intron matched exactly with a previously annotated intron.

### Variant calling and evaluation

Sequence variants in the RNA-seq data were analyzed using GATK’s Unified Genotyper (v2.1.13) [26, 27]. Additionally, the Unified Genotyper was used to analyze variant calls at positions identified by the RNA-seq data using reference genome resequencing data from Rebolledo-Mendez *et al*. 2015 [10]. Based on the recommendations in the GATK manual, the following hard filters were applied to both sets with custom scripts (SNPs: QD < 2.0, MQ < 40.0, FS > 60.0, HaplotypeScore > 13.0 (genomic data only), MQRankSum < -12.5, ReadPosRankSum < -8.0; Indels: QD < 2.0, ReadPosRankSum < -20.0, FS > 200.0). Identified variants were compared with existing annotation with custom scripts versus the “Equus caballus Short Variation (SNPs and Indels)” data available in Ensembl 71 Biomart. Variants were also compared to the vcf files for the five breeds in Orlando *et al*. 2013 [3]. The effects of the variants on transcripts and their ORFs were evaluated by first creating fasta sequences of gtf format transcripts with the reference genome fasta file and Cufflinks gffread script. Custom scripts were then used to make an additional fasta format sequence per variant. For variant containing transcripts, the original and altered sequences were then evaluated by EMBOSS v6.5.7 [28] ORF analyses to identify the ORF lengths of the original and variant introduced transcript sequences.

## Supporting Information

S1 Dataset Variants that extend ORF found in both genomic (homozygous) and transcriptomic data. (TXT)

S2 Dataset Tissues and horses used to create the RNA pool, including Agilent Bioanalyzer trace results. (PDF)

S1 Fig BLAST thresholds for 1, 2, or multi-exon transcripts. (PDF)

## References

[1] Wade CM, Giulotto E, Sigurdsson S, Zoli M, Gnerre S, Imsland F, et al Genome sequence, comparative analysis, and population genetics of the domestic horse. Science. 2009;326(5964):865–867. 10.1126/science.1178158 19892987 PMC3785132

[2] Tang F, Barbacioru C, Wang Y, Nordman E, Lee C, Xu N, et al mRNA-Seq whole-transcriptome analysis of a single cell. Nat Methods. 2009;6:377–382. 10.1038/nmeth.1315 19349980

[3] Orlando L, Ginolhac A, Zhang G, Froese D, Albrechtsen A, Stiller M, et al Recalibrating Equus evolution using the genome sequence of an early Middle Pleistocene horse. Nature. 2013;499(7456):74–78. 10.1038/nature12323 23803765

[4] Doan R, Cohen ND, Sawyer J, Ghaffari N, Johnson CD, Dindot SV. Whole-genome sequencing and genetic variant analysis of a Quarter Horse mare. BMC Genomics. 2012;13:78 10.1186/1471-2164-13-78 22340285 PMC3309927

[5] Metzger J, Tonda R, Beltran S, Agueda L, Gut M, Distl O. Next generation sequencing gives an insight into the characteristics of highly selected breeds versus non-breed horses in the course of domestication. BMC Genomics. 2014;15:562 10.1186/1471-2164-15-562 24996778 PMC4097168

[6] Coleman SJ, Zeng Z, Wang K, Luo S, Khrebtukova I, Mienaltowski MJ, et al Structural annotation of equine protein-coding genes determined by mRNA sequencing. Anim Genet. 2010;41 Suppl 2:121–30. 10.1111/j.1365-2052.2010.02118.x 21070285

[7] Rustici G, Kolesnikov N, Brandizi M, Burdett T, Dylag M, Emam I, et al ArrayExpress update-trends in database growth and links to data analysis tools. Nucleic Acids Res. 2013;41(Database issue):D987–990. 10.1093/nar/gks1174 23193272 PMC3531147

[8] Flicek P, Ahmed I, Amode MR, Barrell D, Beal K, Brent S, et al Ensembl 2013. Nucleic Acids Res. 2013;41(Database issue):48–55. 10.1093/nar/gks1236 PMC3531136 23203987

[9] Altschul SF, Gish W, Miller W, Myers EW, Lipman DJ. Basic local alignment search tool. J Mol Biol. 1990;215(3):403–10. 10.1016/S0022-2836(05)80360-2 2231712

[10] Rebolledo-Mendez J, Hestand MS, Coleman SJ, Zeng Z, Orlando L, MacLeod JN, et al Comparison of the Equine Reference Sequence with its Sanger Source Data and New Illumina Reads. PLoS ONE. 2015; 10(6). 10.1371/journal.pone.0126852 PMC4479572 26107638

[11] Robinson JT, Thorvaldsdottir H, Winckler W, Guttman M, Lander ES, Getz G, et al Integrative genomics viewer. Nat Biotechnol. 2011;29(1):24–26. 10.1038/nbt.1754 21221095 PMC3346182

[12] Ladoukakis E, Pereira V, Magny EG, Eyre-Walker A, Couso JP. Hundreds of putatively functional small open reading frames in Drosophila. Genome Biol. 2011;12(11):R118 10.1186/gb-2011-12-11-r118 22118156 PMC3334604

[13] Magny EG, Pueyo JI, Pearl FM, Cespedes MA, Niven JE, Bishop SA, et al Conserved regulation of cardiac calcium uptake by peptides encoded in small open reading frames. Science. 2013;341(6150):1116–1120. 10.1126/science.1238802 23970561

[14] Schwarzbauer JE, DeSimone DW. Fibronectins, their fibrillogenesis, and in vivo functions. Cold Spring Harb Perspect Biol. 2011;3(7). 10.1101/cshperspect.a005041 21576254 PMC3119908

[15] MacLeod JN, Burton-Wurster N, Gu DN, Lust G. Fibronectin mRNA splice variant in articular cartilage lacks bases encoding the V, III-15, and I-10 protein segments. J Biol Chem. 1996;271(31):18954–18960. 10.1074/jbc.271.31.18954 8702559

[16] Chomczynski P, Sacchi N. Single-step method of RNA isolation by acid guanidinium thiocyanate-phenol-chloroform extraction. Anal Biochem. 1987;162(1):156–159. 10.1016/0003-2697(87)90021-2 2440339

[17] Lambert JD, Chan XY, Spiecker B, Sweet HC. Characterizing the embryonic transcriptome of the snail Ilyanassa. Integr Comp Biol. 2010;50(5):768–777. 10.1093/icb/icq121 21558239

[18] Hestand MS, Klingenhoff A, Scherf M, Ariyurek Y, Ramos Y, van Workum W, et al Tissue-specific transcript annotation and expression profiling with complementary next-generation sequencing technologies. Nucleic Acids Res. 2010;38(16):e165 10.1093/nar/gkq602 20615900 PMC2938216

[19] Wang K, Singh D, Zeng Z, Coleman SJ, Huang Y, Savich GL, et al MapSplice: accurate mapping of RNA-seq reads for splice junction discovery. Nucleic Acids Res. 2010;38(18):e178 10.1093/nar/gkq622 20802226 PMC2952873

[20] Trapnell C, Williams BA, Pertea G, Mortazavi A, Kwan G, van Baren MJ, et al Transcript assembly and quantification by RNA-Seq reveals unannotated transcripts and isoform switching during cell differentiation. Nat Biotechnol. 2010;28(5):511–515. 10.1038/nbt.1621 20436464 PMC3146043

[21] Langmead B, Trapnell C, Pop M, Salzberg SL. Ultrafast and memory-efficient alignment of short DNA sequences to the human genome. Genome Biol. 2009;10(3):R25 10.1186/gb-2009-10-3-r25 19261174 PMC2690996

[22] Kent WJ. BLAT-the BLAST-like alignment tool. Genome Res. 2002;12(4):656–664. 10.1101/gr.229202 11932250 PMC187518

[23] Kent WJ, Sugnet CW, Furey TS, Roskin KM, Pringle TH, Zahler AM, et al The human genome browser at UCSC. Genome Res. 2002;12(6):996–1006. 10.1101/gr.229102 12045153 PMC186604

[24] Karolchik D, Hinrichs AS, Furey TS, Roskin KM, Sugnet CW, Haussler D, et al The UCSC Table Browser data retrieval tool. Nucleic Acids Res. 2004;32(Database issue):D493–496. 10.1093/nar/gkh103 14681465 PMC308837

[25] Fujita PA, Rhead B, Zweig AS, Hinrichs AS, Karolchik D, Cline MS, et al The UCSC Genome Browser database: update 2011. Nucleic Acids Res. 2011;39(Database issue):D876–882. 10.1093/nar/gkq963 20959295 PMC3242726

[26] McKenna A, Hanna M, Banks E, Sivachenko A, Cibulskis K, Kernytsky A, et al The Genome Analysis Toolkit: a MapReduce framework for analyzing next-generation DNA sequencing data. Genome Res. 2010;20(9):1297–303. 10.1101/gr.107524.110 20644199 PMC2928508

[27] DePristo MA, Banks E, Poplin R, Garimella KV, Maguire JR, Hartl C, et al A framework for variation discovery and genotyping using next-generation DNA sequencing data. Nat Genet. 2011;43(5):491–8. 10.1038/ng.806 21478889 PMC3083463

[28] Rice P, Longden I, Bleasby A. EMBOSS: the European Molecular Biology Open Software Suite. Trends Genet. 2000;16(6):276–7. 10.1016/S0168-9525(00)02024-2 10827456

