# Supplementary material for: Annotation of the Protein Coding Regions of the Equine Genome
Source: PLoS One. 2015 Jun 24;10(6):e0124375. doi: 10.1371/journal.pone.0124375 (PMC4481266; doi:10.1371/journal.pone.0124375)
Supplement: S2 Dataset — (PDF) [file pone.0124375.s002.pdf]

Supplemental File (Hestand et al. 2014 "Annotation of the Protein Coding Regions of the Equine Genome")

Samples: (equal relative contribution to RNA pool)

Adipose Tissue, H1
Adrenal Cortex, H1
Adrenal Medulla, H1
Aorta, H1
Articular Cartilage (foal), H2
Bladder, H1
Bone, H1
Bone Marrow, H1
Cecum, H1
Cerebrum, H1
Cornea, H1
Embryo (whole embryo, 34 days), H3
Endometrium (pregnant day 16), H4
Endometrium (pregnant day 50), H5
Epididymus, H6
Hoof (germinal epithelium), H1
Kidney, H1
Large Intestine, H1
Liver, H1
Lung, H1
Lymph Node, H1
Lymphocytes (activated), H7
Muscle (cardiac), H1
Muscle (skeletal, tongue), H6
Muscle (skeletal), H1
Muscle (smooth), H1
Ovary, H1
Pancreas, H8
Pituitary (anterior), H1
Pituitary (posterior), H1
Placental Villous, H9
Retina, H1
Salivary Gland, H8
Skin (full thickness), H1
Spinal Cord, H1
Spinal Root Ganglia, H1
Spleen (red pulp), H1
Spleen (white pulp), H1
Stomach, H1
Synovial Membrane, H10
Tendon (super digital flexor, foal), H11
Testes, H6
Thymus, H1
Vena Cava, H1

H numbers indicate the horse from which the sample originated. Samples were from sexually mature young adult horses unless otherwise indicted.

Pooled Sample RNA Quantitation and Purity (Nanodrop)

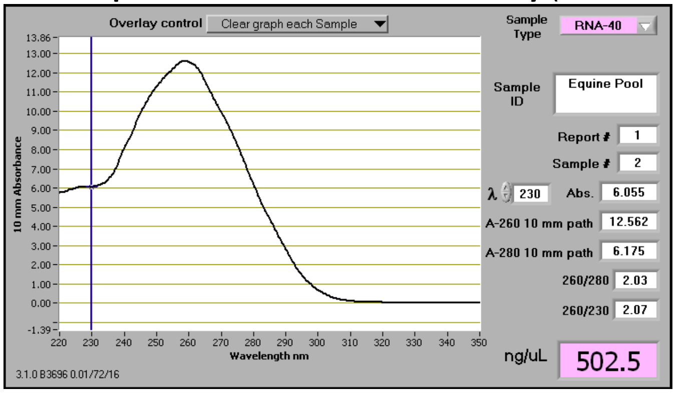

Pooled Sample RNA Integrity (Agilent Bioanalyzer)

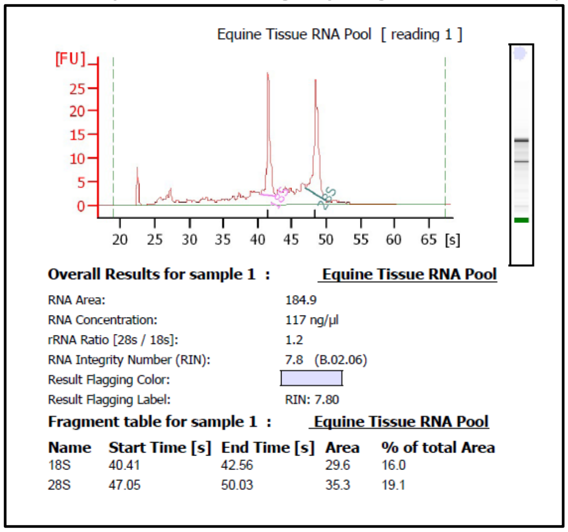

RNA Quantitation, Purity, and Integrity Data for Individual Samples

Adipose Tissue

Integrity:

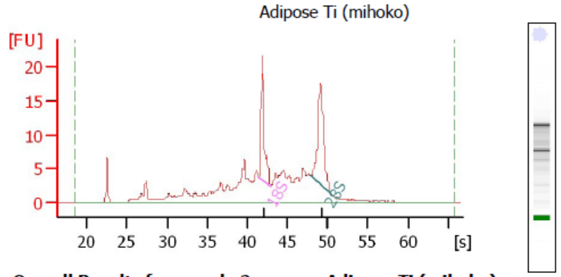

Overall Results for sample 2 : Adipose Ti (mihoko)

RNA Area:	194.1
RNA Concentration:	150 ng/µl
rRNA Ratio [28s / 18s]:	1.2
RNA Integrity Number (RIN):	6.8 (B.02.06)
Result Flagging Color:	
Result Flagging Label:	RIN: 6.80

Fragment table for sample 2 : Adipose Ti (mihoko)

Name	Start Time [s]	End Time [s]	Area	% of total Area
18S	41.38	42.85	21.2	10.9
28S	47.96	50.63	26.2	13.5

Quantitation and Purity:

260/280: 2.08
260/230: 1.94
Concentration (ng/µl): 308

Adrenal Medulla

Integrity:

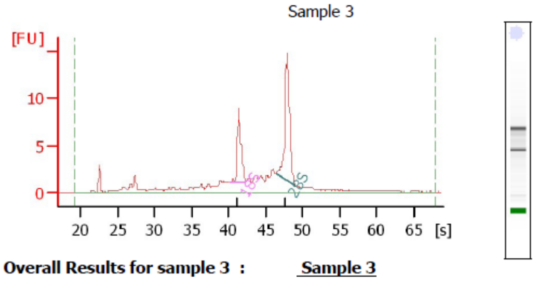

Overall Results for sample 3 : Sample 3

RNA Area:	95.5
RNA Concentration:	137 ng/µl
rRNA Ratio [28s / 18s]:	1.9
RNA Integrity Number (RIN):	8.4 (B.02.06)
Result Flagging Color:	
Result Flagging Label:	RIN: 8.40

Fragment table for sample 3 : Sample 3

Name	Start Time [s]	End Time [s]	Area	% of total Area
18S	40.13	42.29	11.2	11.7
28S	46.38	49.09	20.8	21.8

Quantitation and Purity:

260/280: 2.02
260/230: 2.17
Concentration (ng/µl): 500

Adrenal Cortex

Integrity:

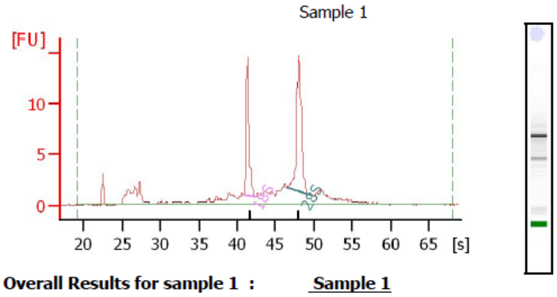

Overall Results for sample 1 : Sample 1

RNA Area:	89.3
RNA Concentration:	128 ng/µl
rRNA Ratio [28s / 18s]:	1.5
RNA Integrity Number (RIN):	8.5 (B.02.06)
Result Flagging Color:	
Result Flagging Label:	RIN: 8.50

Fragment table for sample 1 : Sample 1

Name	Start Time [s]	End Time [s]	Area	% of total Area
18S	40.90	42.38	13.9	15.6
28S	46.53	49.69	20.6	23.0

Quantitation and Purity:

260/280: 1.92
260/230: 2.17
Concentration (ng/µl): 500

Aorta

Integrity:

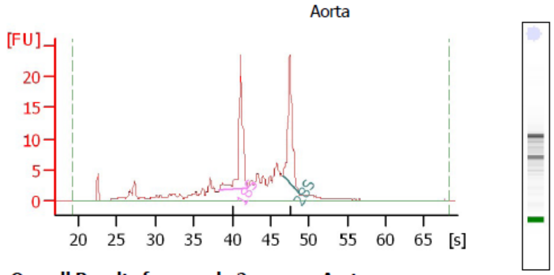

Overall Results for sample 2 : Aorta

RNA Area:	170.8
RNA Concentration:	206 ng/µl
rRNA Ratio [28s / 18s]:	0.9
RNA Integrity Number (RIN):	8.1 (B.02.06)
Result Flagging Color:	
Result Flagging Label:	RIN: 8.10

Fragment table for sample 2 : Aorta

Name	Start Time [s]	End Time [s]	Area	% of total Area
18S	38.32	42.08	32.1	18.8
28S	46.53	48.71	27.6	16.1

Quantitation and Purity:

260/280: 1.90
260/230: 2.07
Concentration (ng/µl): 500

Articular Cartilage (foal)

Integrity:

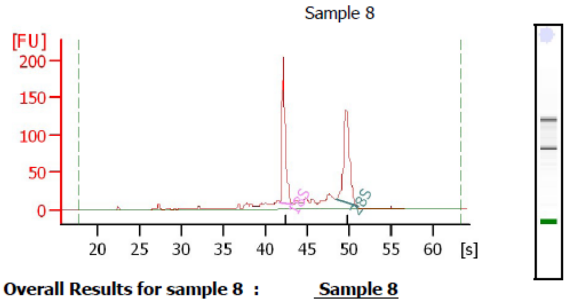

Overall Results for sample 8 : Sample 8

RNA Area:	786.1
RNA Concentration:	239 ng/µl
rRNA Ratio [28s / 18s]:	1.2
RNA Integrity Number (RIN):	8.8 (B.02.06)
Result Flagging Color:	
Result Flagging Label:	RIN: 8.80

Fragment table for sample 8 : Sample 8

Name	Start Time [s]	End Time [s]	Area	% of total Area
18S	41.83	43.35	184.7	23.5
28S	48.54	51.11	214.4	27.3

Quantitation and Purity:

260/280: 2.07

260/230: 2.14

Concentration (ng/µl): 199.2

Bone

Integrity:

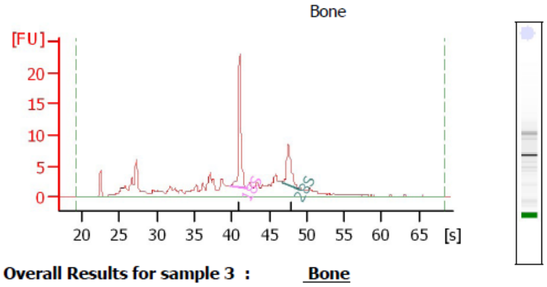

Overall Results for sample 3 : Bone

RNA Area:	148.0
RNA Concentration:	178 ng/µl
rRNA Ratio [28s / 18s]:	0.4
RNA Integrity Number (RIN):	6.7 (B.02.06)
Result Flagging Color:	
Result Flagging Label:	RIN: 6.70

Fragment table for sample 3 : Bone

Name	Start Time [s]	End Time [s]	Area	% of total Area
18S	39.65	41.98	21.5	14.5
28S	46.73	49.16	9.1	6.1

Quantitation and Purity:

260/280: 1.97

260/230: 2.08

Concentration (ng/µl): 500

Bladder

Integrity:

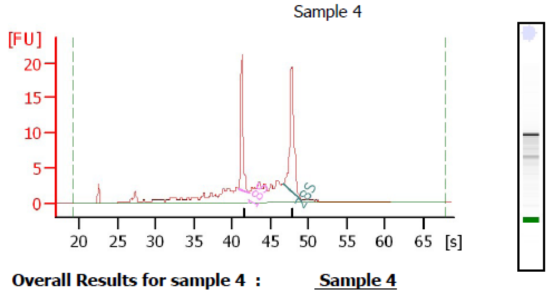

Overall Results for sample 4 : Sample 4

RNA Area:	113.6
RNA Concentration:	163 ng/µl
rRNA Ratio [28s / 18s]:	1.3
RNA Integrity Number (RIN):	7.5 (B.02.06)
Result Flagging Color:	
Result Flagging Label:	RIN: 7.50

Fragment table for sample 4 : Sample 4

Name	Start Time [s]	End Time [s]	Area	% of total Area
18S	40.86	42.14	17.0	14.9
28S	46.72	49.04	22.5	19.8

Quantitation and Purity:

260/280: 2.05

260/230: 1.87

Concentration (ng/µl): 479

Bone Marrow

Integrity:

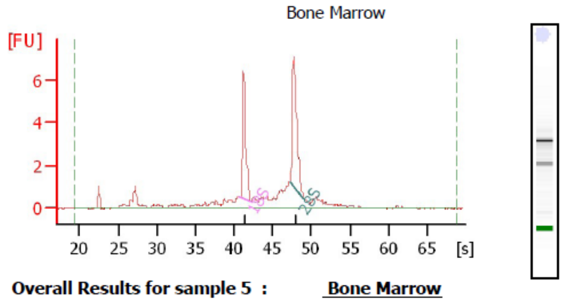

Overall Results for sample 5 : Bone Marrow

RNA Area:	38.7
RNA Concentration:	181 ng/µl
rRNA Ratio [28s / 18s]:	1.2
RNA Integrity Number (RIN):	8.4 (A.01.01)
Result Flagging Color:	
Result Flagging Label:	RIN: 8.40

Fragment table for sample 5 : Bone Marrow

Name	Start Time [s]	End Time [s]	Area	% of total Area
18S	40.76	42.26	7.0	18.0
28S	47.25	49.04	8.5	22.0

Quantitation and Purity:

260/280: 2.02

260/230: 2.2

Concentration (ng/µl): 500

Cecum

Integrity:

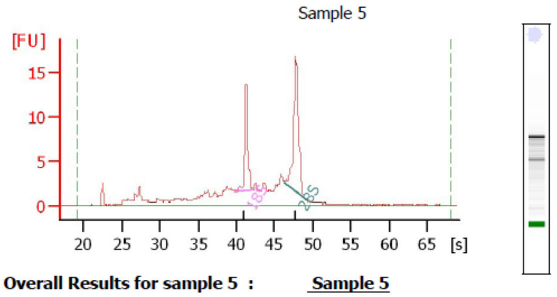

Overall Results for sample 5 : **Sample 5**

RNA Area: 123.3
RNA Concentration: 177 ng/µl
rRNA Ratio [28s / 18s]: 1.7
RNA Integrity Number (RIN): 7.6 (B.02.06)
Result Flagging Color:
Result Flagging Label: RIN: 7.60

Fragment table for sample 5 : **Sample 5**

Name	Start Time [s]	End Time [s]	Area	% of total Area
18S	39.82	42.19	13.4	10.8
28S	46.33	49.24	23.0	18.6

Quantitation and Purity:
260/280: 2.00
260/230: 2.06
Concentration (ng/µl): 500

Cornea

Integrity:

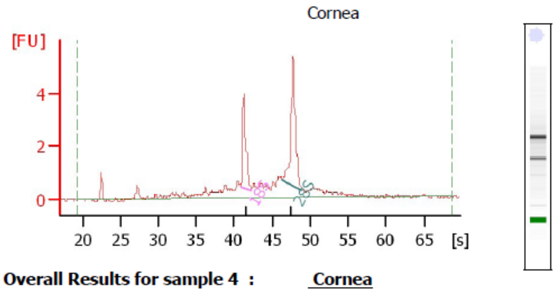

Overall Results for sample 4 : **Cornea**

RNA Area: 31.5
RNA Concentration: 147 ng/µl
rRNA Ratio [28s / 18s]: 1.7
RNA Integrity Number (RIN): 7.9 (A.01.01)
Result Flagging Color:
Result Flagging Label: RIN: 7.90

Fragment table for sample 4 : **Cornea**

Name	Start Time [s]	End Time [s]	Area	% of total Area
18S	40.87	42.26	4.2	13.3
28S	46.34	48.93	6.9	22.0

Quantitation and Purity:
260/280: 2.07
260/230: 2.24
Concentration (ng/µl): 500

Cerebrum

Integrity:

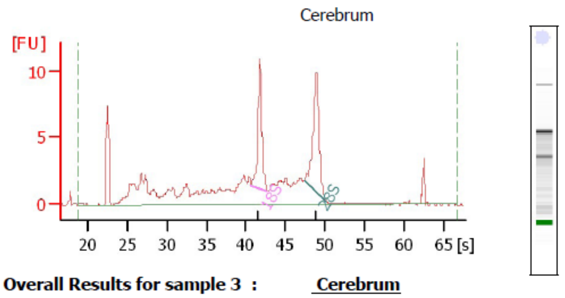

Overall Results for sample 3 : **Cerebrum**

RNA Area: 116.7
RNA Concentration: 68 ng/µl
rRNA Ratio [28s / 18s]: 1.3
RNA Integrity Number (RIN): 7.2 (B.02.06)
Result Flagging Color:
Result Flagging Label: RIN: 7.20

Fragment table for sample 3 : **Cerebrum**

Name	Start Time [s]	End Time [s]	Area	% of total Area
18S	40.69	42.77	13.1	11.2
28S	47.42	50.27	16.5	14.1

Quantitation and Purity:
260/280: 2.04
260/230: 2.15
Concentration (ng/µl): 500

Embryo (whole embryo, 34 days)

Integrity:

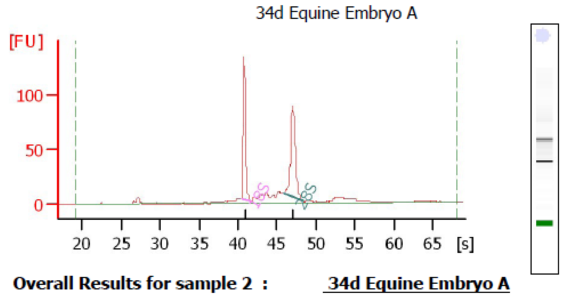

Overall Results for sample 2 : **34d Equine Embryo A**

RNA Area: 447.9
RNA Concentration: 359 ng/µl
rRNA Ratio [28s / 18s]: 1.0
RNA Integrity Number (RIN): 9.6 (B.02.06)
Result Flagging Color:
Result Flagging Label: RIN: 9.60

Fragment table for sample 2 : **34d Equine Embryo A**

Name	Start Time [s]	End Time [s]	Area	% of total Area
18S	40.36	41.60	125.4	28.0
28S	45.74	48.36	130.9	29.2

Quantitation and Purity:
260/280: 2.00
260/230: 2.20
Concentration (ng/µl): 1249.3

Endometrium (pregnant day 16)

Integrity:

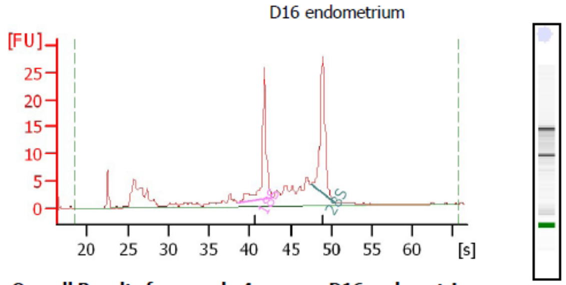

Overall Results for sample 4 : **D16 endometrium**

RNA Area:	181.6
RNA Concentration:	140 ng/µl
rRNA Ratio [28s / 18s]:	1.0
RNA Integrity Number (RIN):	9 (B.02.06)
Result Flagging Color:	
Result Flagging Label:	RIN:9

Fragment table for sample 4 : **D16 endometrium**

Name	Start Time [s]	End Time [s]	Area	% of total Area
18S	38.63	42.81	36.6	20.2
28S	47.52	50.42	37.4	20.6

Quantitation and Purity:

260/280: 1.98

260/230: 2.31

Concentration (ng/µl): 484

Epididymus

Integrity:

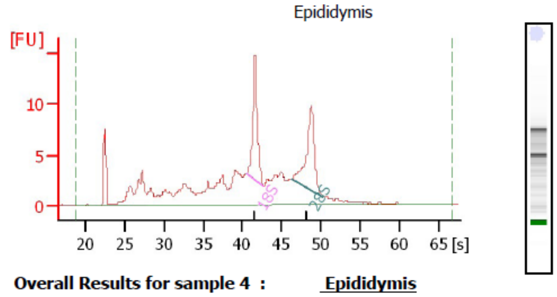

Overall Results for sample 4 : **Epididymis**

RNA Area:	177.8
RNA Concentration:	104 ng/µl
rRNA Ratio [28s / 18s]:	1.0
RNA Integrity Number (RIN):	6.9 (B.02.06)
Result Flagging Color:	
Result Flagging Label:	RIN: 6.90

Fragment table for sample 4 : **Epididymis**

Name	Start Time [s]	End Time [s]	Area	% of total Area
18S	40.55	42.68	19.6	11.0
28S	46.31	50.27	20.2	11.4

Quantitation and Purity:

260/280: 1.88

260/230: 2.07

Concentration (ng/µl): 500

Endometrium (pregnant day 50)

Integrity:

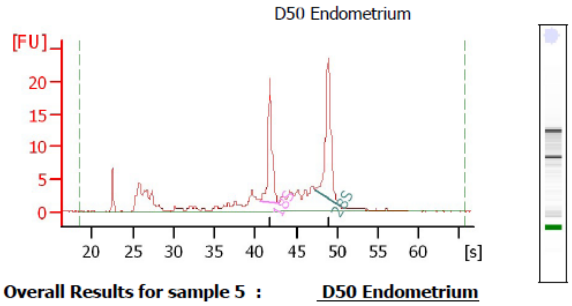

Overall Results for sample 5 : **D50 Endometrium**

RNA Area:	165.2
RNA Concentration:	127 ng/µl
rRNA Ratio [28s / 18s]:	1.3
RNA Integrity Number (RIN):	8.4 (B.02.06)
Result Flagging Color:	
Result Flagging Label:	RIN: 8.40

Fragment table for sample 5 : **D50 Endometrium**

Name	Start Time [s]	End Time [s]	Area	% of total Area
18S	40.62	42.81	26.9	16.3
28S	47.33	50.47	33.9	20.5

Quantitation and Purity:

260/280: 1.97

260/230: 2.31

Concentration (ng/µl): 465.6

Hoof (germinal epithelium)

Integrity:

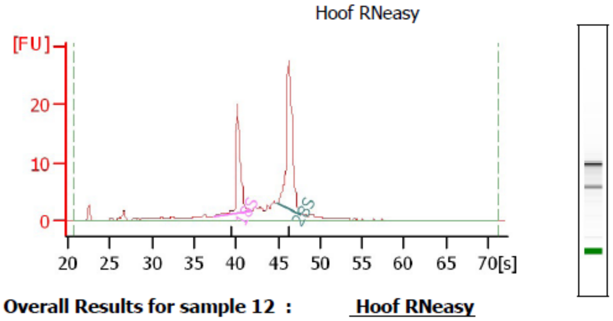

Overall Results for sample 12 : **Hoof RNeasy**

RNA Area:	144.9
RNA Concentration:	187 ng/µl
rRNA Ratio [28s / 18s]:	1.7
RNA Integrity Number (RIN):	9.5 (B.02.06)

Fragment table for sample 12 : **Hoof RNeasy**

Name	Start Time [s]	End Time [s]	Area	% of total Area
18S	37.26	41.75	28.0	19.3
28S	44.90	47.68	46.6	32.1

Quantitation and Purity:

260/280: 1.94

260/230: 2.17

Concentration (ng/µl): 500

Kidney

Integrity:

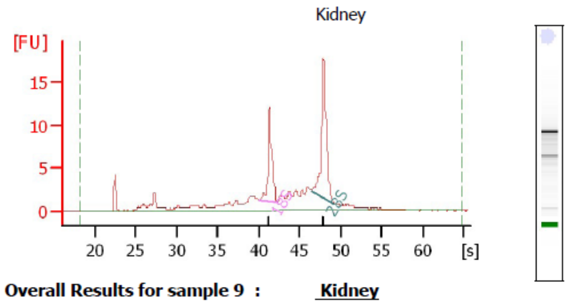

Overall Results for sample 9 : Kidney

RNA Area:	108.1
RNA Concentration:	99 ng/µl
rRNA Ratio [28s / 18s]:	1.5
RNA Integrity Number (RIN):	8.3 (B.02.06)
Result Flagging Color:	
Result Flagging Label:	RIN: 8.30

Fragment table for sample 9 : Kidney

Name	Start Time [s]	End Time [s]	Area	% of total Area
18S	40.07	42.32	15.1	14.0
28S	46.50	49.27	23.1	21.4

Quantitation and Purity:
260/280: 2.02
260/230: 2.05
Concentration (ng/µl): 500

Liver

Integrity:

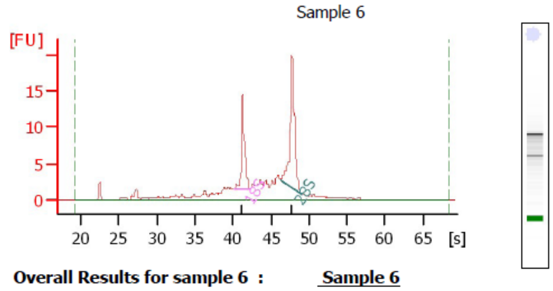

Overall Results for sample 6 : Sample 6

RNA Area:	112.9
RNA Concentration:	162 ng/µl
rRNA Ratio [28s / 18s]:	1.6
RNA Integrity Number (RIN):	8.3 (B.02.06)
Result Flagging Color:	
Result Flagging Label:	RIN: 8.30

Fragment table for sample 6 : Sample 6

Name	Start Time [s]	End Time [s]	Area	% of total Area
18S	40.24	42.27	16.3	14.4
28S	46.29	49.11	26.6	23.6

Quantitation and Purity:
260/280: 2.11
260/230: 1.89
Concentration (ng/µl): 500

Large Intestine

Integrity:

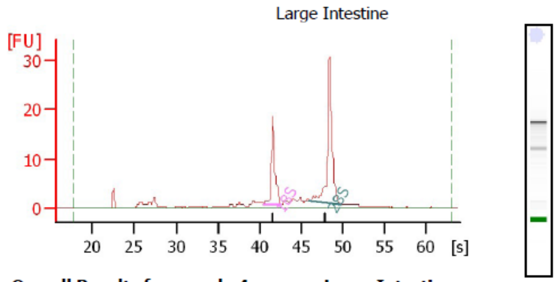

Overall Results for sample 4 : Large Intestine

RNA Area:	115.4
RNA Concentration:	105 ng/µl
rRNA Ratio [28s / 18s]:	1.9
RNA Integrity Number (RIN):	9.4 (B.02.06)
Result Flagging Color:	
Result Flagging Label:	RIN: 9.40

Fragment table for sample 4 : Large Intestine

Name	Start Time [s]	End Time [s]	Area	% of total Area
18S	40.48	42.63	20.6	17.9
28S	45.96	49.79	40.0	34.7

Quantitation and Purity:
260/280: 2.07
260/230: 2.14
Concentration (ng/µl): 1000

Lung

Integrity:

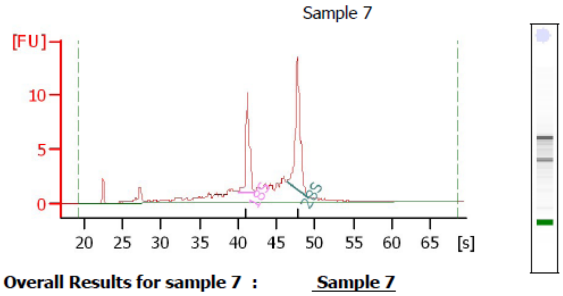

Overall Results for sample 7 : Sample 7

RNA Area:	82.1
RNA Concentration:	118 ng/µl
rRNA Ratio [28s / 18s]:	1.5
RNA Integrity Number (RIN):	8.1 (B.02.06)
Result Flagging Color:	
Result Flagging Label:	RIN: 8.10

Fragment table for sample 7 : Sample 7

Name	Start Time [s]	End Time [s]	Area	% of total Area
18S	40.08	42.22	12.3	15.0
28S	46.29	49.27	18.6	22.6

Quantitation and Purity:
260/280: 1.94
260/230: 2.29
Concentration (ng/µl): 500

Lymph Node

Integrity:

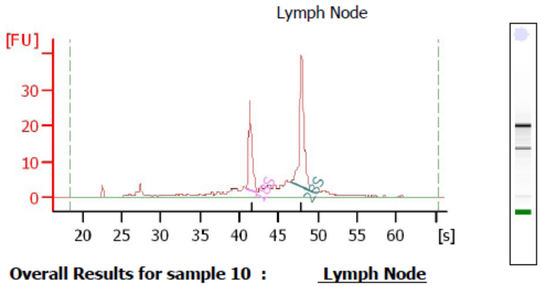

Overall Results for sample 10 : **Lymph Node**

RNA Area:	196.7
RNA Concentration:	179 ng/µl
rRNA Ratio [28s / 18s]:	1.9
RNA Integrity Number (RIN):	8.5 (B.02.06)
Result Flagging Color:	
Result Flagging Label:	RIN: 8.50

Fragment table for sample 10 : **Lymph Node**

Name	Start Time [s]	End Time [s]	Area	% of total Area
18S	40.88	42.30	27.0	13.7
28S	46.42	49.36	50.3	25.6

Quantitation and Purity:

260/280: 2.01

260/230: 2.12

Concentration (ng/µl): 410

Muscle (cardiac)

Integrity:

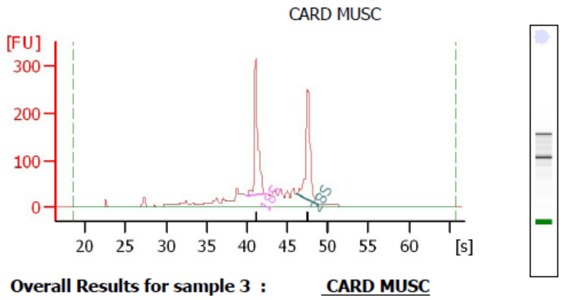

Overall Results for sample 3 : **CARD MUSC**

RNA Area:	1,564.3
RNA Concentration:	649 ng/µl
rRNA Ratio [28s / 18s]:	0.9
RNA Integrity Number (RIN):	8 (B.02.06)
Result Flagging Color:	
Result Flagging Label:	RIN:8

Fragment table for sample 3 : **CARD MUSC**

Name	Start Time [s]	End Time [s]	Area	% of total Area
18S	39.99	42.19	349.5	22.3
28S	46.10	48.72	298.1	19.1

Quantitation and Purity:

260/280: 2.11

260/230: 2.24

Concentration (ng/µl): 1072

Lymphocytes (activated)

Integrity:

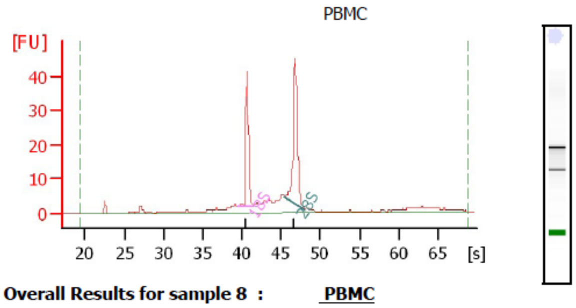

Overall Results for sample 8 : **PBMC**

RNA Area:	175.3
RNA Concentration:	84 ng/µl
rRNA Ratio [28s / 18s]:	1.7
RNA Integrity Number (RIN):	9.2 (B.02.06)
Result Flagging Color:	
Result Flagging Label:	RIN: 9.20

Fragment table for sample 8 : **PBMC**

Name	Start Time [s]	End Time [s]	Area	% of total Area
18S	39.56	41.51	32.3	18.4
28S	45.50	48.04	55.1	31.4

Quantitation and Purity:

260/280: 1.99

260/230: 2.00

Concentration (ng/µl): 500

Muscle (skeletal, tongue)

Integrity:

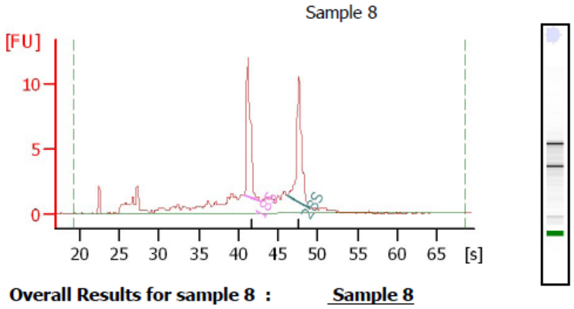

Overall Results for sample 8 : **Sample 8**

RNA Area:	79.2
RNA Concentration:	114 ng/µl
rRNA Ratio [28s / 18s]:	0.9
RNA Integrity Number (RIN):	7.6 (B.02.06)
Result Flagging Color:	
Result Flagging Label:	RIN: 7.60

Fragment table for sample 8 : **Sample 8**

Name	Start Time [s]	End Time [s]	Area	% of total Area
18S	40.68	42.81	15.2	19.2
28S	46.09	49.07	14.2	17.9

Quantitation and Purity:

260/280: 2.08

260/230: 2.18

Concentration (ng/µl): 500

Muscle (skeletal)

Integrity:

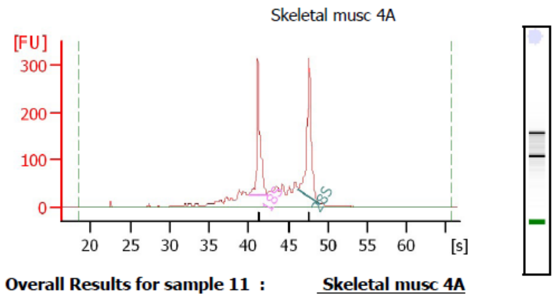

Overall Results for sample 11 : **Skeletal musc 4A**

RNA Area:	1,659.7
RNA Concentration:	469 ng/µl
rRNA Ratio [28s / 18s]:	1.1
RNA Integrity Number (RIN):	8.1 (B.02.06)
Result Flagging Color:	
Result Flagging Label:	RIN: 8.10

Fragment table for sample 11 : **Skeletal musc 4A**

Name	Start Time [s]	End Time [s]	Area	% of total Area
18S	40.10	42.29	324.5	19.5
28S	46.28	48.85	355.9	21.4

Quantitation and Purity:

260/280: 2.08

260/230: 2.11

Concentration (ng/µl): 492.28

Ovary

Integrity:

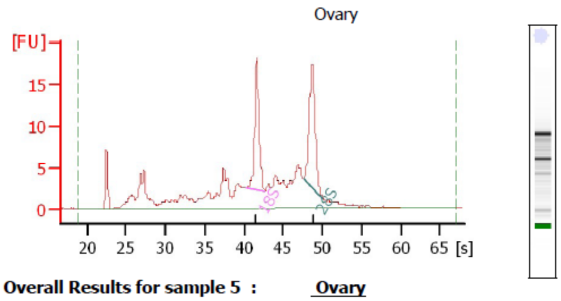

Overall Results for sample 5 : **Ovary**

RNA Area:	199.3
RNA Concentration:	117 ng/µl
rRNA Ratio [28s / 18s]:	1.1
RNA Integrity Number (RIN):	7.2 (B.02.06)
Result Flagging Color:	
Result Flagging Label:	RIN: 7.20

Fragment table for sample 5 : **Ovary**

Name	Start Time [s]	End Time [s]	Area	% of total Area
18S	40.38	42.67	24.7	12.4
28S	47.58	50.05	26.7	13.4

Quantitation and Purity:

260/280: 1.97

260/230: 2.19

Concentration (ng/µl): 500

Muscle (smooth)

Integrity:

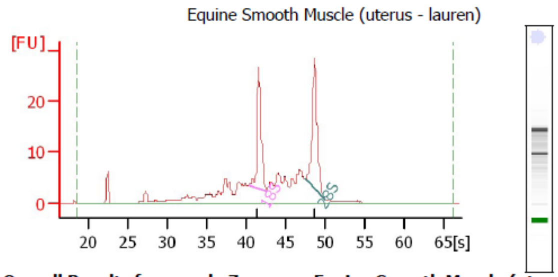

Overall Results for sample 7 : **Equine Smooth Muscle (uterus - l**

RNA Area:	217.5
RNA Concentration:	168 ng/µl
rRNA Ratio [28s / 18s]:	1.2
RNA Integrity Number (RIN):	7.4 (B.02.06)
Result Flagging Color:	
Result Flagging Label:	RIN: 7.40

Fragment table for sample 7 : **Equine Smooth Muscle (uterus - la**

Name	Start Time [s]	End Time [s]	Area	% of total Area
18S	40.44	42.60	31.8	14.6
28S	47.59	50.09	37.4	17.2

Quantitation and Purity:

260/280: 2.08

260/230: 2.20

Concentration (ng/µl): 472

Pancreas

Integrity:

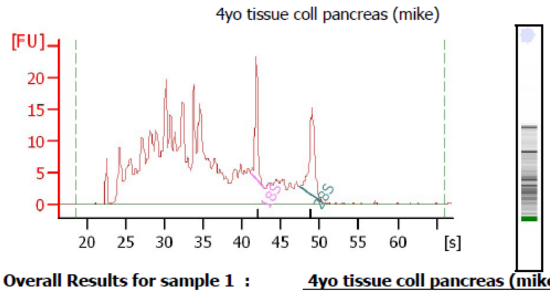

Overall Results for sample 1 : **4yo tissue coll pancreas (mike)**

RNA Area:	522.4
RNA Concentration:	402 ng/µl
rRNA Ratio [28s / 18s]:	1.2
RNA Integrity Number (RIN):	4.8 (B.02.06)
Result Flagging Color:	
Result Flagging Label:	RIN: 4.80

Fragment table for sample 1 : **4yo tissue coll pancreas (mike)**

Name	Start Time [s]	End Time [s]	Area	% of total Area
18S	41.03	42.89	18.6	3.6
28S	47.39	50.31	21.5	4.1

Quantitation and Purity:

260/280: 2.09

260/230: 2.20

Concentration (ng/µl): 1779

Pituitary (anterior)

Integrity:

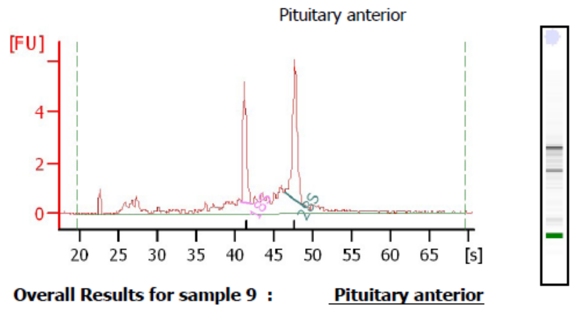

Overall Results for sample 9 : Pituitary anterior

RNA Area:	37.5
RNA Concentration:	175 ng/µl
rRNA Ratio [28s / 18s]:	1.6
RNA Integrity Number (RIN):	8.1 (A.01.01)
Result Flagging Color:	
Result Flagging Label:	RIN: 8.10

Fragment table for sample 9 : Pituitary anterior

Name	Start Time [s]	End Time [s]	Area	% of total Area
18S	40.71	42.07	5.0	13.3
28S	46.36	49.04	7.8	20.8

Quantitation and Purity:

260/280: 2.02

260/230: 2.27

Concentration (ng/µl): 500

Placental Villous

Integrity:

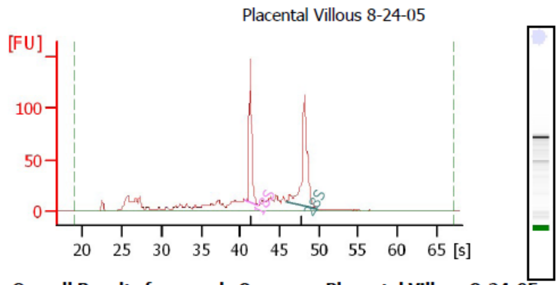

Overall Results for sample 8 : Placental Villous 8-24-05

RNA Area:	735.8
RNA Concentration:	446 ng/µl
rRNA Ratio [28s / 18s]:	1.5
RNA Integrity Number (RIN):	7.8 (B.02.06)
Result Flagging Color:	
Result Flagging Label:	RIN: 7.80

Fragment table for sample 8 : Placental Villous 8-24-05

Name	Start Time [s]	End Time [s]	Area	% of total Area
18S	40.71	42.22	113.1	15.4
28S	45.93	49.82	172.1	23.4

Quantitation and Purity:

260/280: 1.94

260/230: 2.22

Concentration (ng/µl): 1018

Pituitary (posterior)

Integrity:

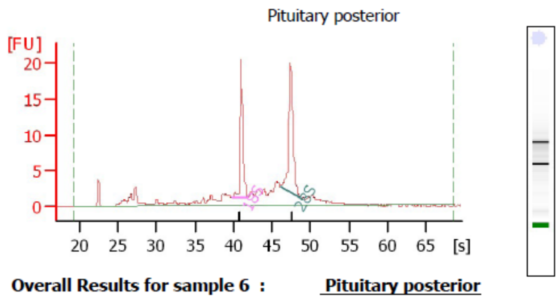

Overall Results for sample 6 : Pituitary posterior

RNA Area:	126.2
RNA Concentration:	152 ng/µl
rRNA Ratio [28s / 18s]:	1.3
RNA Integrity Number (RIN):	8.2 (B.02.06)
Result Flagging Color:	
Result Flagging Label:	RIN: 8.20

Fragment table for sample 6 : Pituitary posterior

Name	Start Time [s]	End Time [s]	Area	% of total Area
18S	39.88	41.92	20.5	16.3
28S	46.04	48.87	26.0	20.6

Quantitation and Purity:

260/280: 2.05

260/230: 2.24

Concentration (ng/µl): 500

Retina

Integrity:

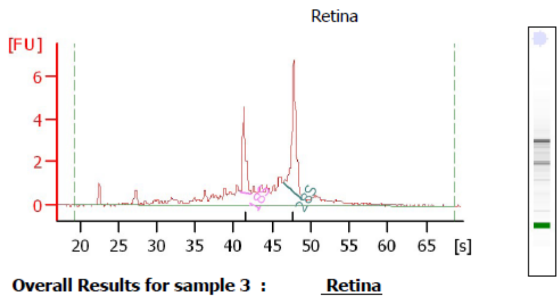

Overall Results for sample 3 : Retina

RNA Area:	43.7
RNA Concentration:	204 ng/µl
rRNA Ratio [28s / 18s]:	1.8
RNA Integrity Number (RIN):	7.7 (A.01.01)
Result Flagging Color:	
Result Flagging Label:	RIN: 7.70

Fragment table for sample 3 : Retina

Name	Start Time [s]	End Time [s]	Area	% of total Area
18S	40.87	42.31	4.5	10.2
28S	46.49	48.88	8.2	18.7

Quantitation and Purity:

260/280: 2.07

260/230: 2.19

Concentration (ng/µl): 500

Salivary Gland

Integrity:

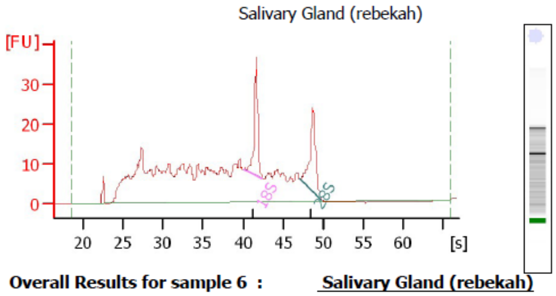

Overall Results for sample 6 : **Salivary Gland (rebekah)**

RNA Area:	589.4
RNA Concentration:	454 ng/µl
rRNA Ratio [28s / 18s]:	1.0
RNA Integrity Number (RIN):	5.4 (B.02.06)
Result Flagging Color:	
Result Flagging Label:	RIN: 5.40

Fragment table for sample 6 : **Salivary Gland (rebekah)**

Name	Start Time [s]	End Time [s]	Area	% of total Area
18S	39.98	42.42	33.7	5.7
28S	47.10	49.92	34.5	5.8

Quantitation and Purity:

260/280: 2.07

260/230: 2.17

Concentration (ng/µl): 3000

Spinal Cord

Integrity:

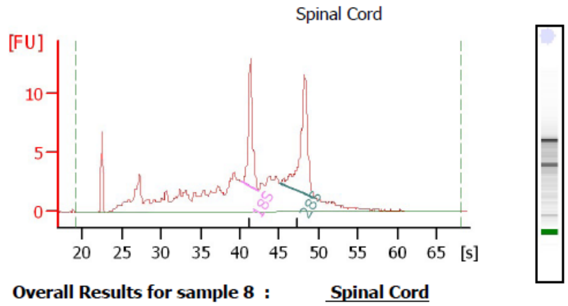

Overall Results for sample 8 : **Spinal Cord**

RNA Area:	162.9
RNA Concentration:	96 ng/µl
rRNA Ratio [28s / 18s]:	1.5
RNA Integrity Number (RIN):	7 (B.02.06)
Result Flagging Color:	
Result Flagging Label:	RIN:7

Fragment table for sample 8 : **Spinal Cord**

Name	Start Time [s]	End Time [s]	Area	% of total Area
18S	40.31	42.38	15.9	9.7
28S	45.10	49.69	23.4	14.4

Quantitation and Purity:

260/280: 2.01

260/230: 2.23

Concentration (ng/µl): 500

Skin (full thickness)

Integrity:

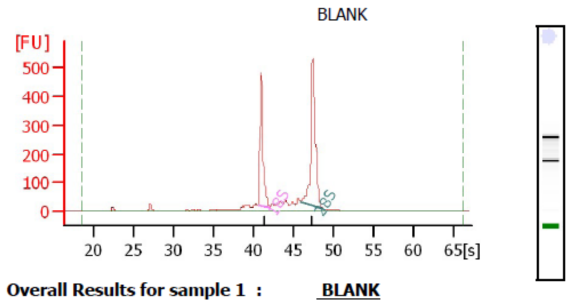

Overall Results for sample 1 : **BLANK**

RNA Area:	1,910.5
RNA Concentration:	792 ng/µl
rRNA Ratio [28s / 18s]:	1.5
RNA Integrity Number (RIN):	9.5 (B.02.06)
Result Flagging Color:	
Result Flagging Label:	RIN: 9.50

Fragment table for sample 1 : **BLANK**

Name	Start Time [s]	End Time [s]	Area	% of total Area
18S	40.63	42.07	477.6	25.0
28S	45.96	48.93	713.9	37.4

Quantitation and Purity:

260/280: 1.95

260/230: 1.89

Concentration (ng/µl): 1000

Spinal Root Ganglia

Integrity:

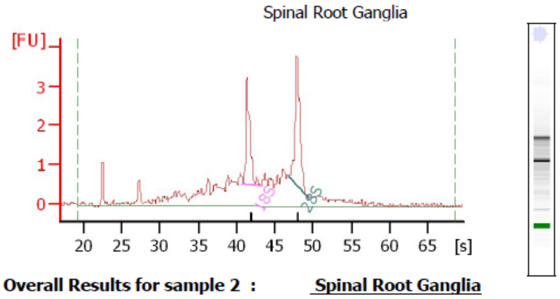

Overall Results for sample 2 : **Spinal Root Ganglia**

RNA Area:	37.8
RNA Concentration:	177 ng/µl
rRNA Ratio [28s / 18s]:	1.2
RNA Integrity Number (RIN):	7.1 (A.01.01)
Result Flagging Color:	
Result Flagging Label:	RIN: 7.10

Fragment table for sample 2 : **Spinal Root Ganglia**

Name	Start Time [s]	End Time [s]	Area	% of total Area
18S	40.83	43.21	4.3	11.2
28S	46.84	49.37	5.1	13.5

Quantitation and Purity:

260/280: 2.07

260/230: 2.16

Concentration (ng/µl): 500

Spleen (red pulp)

Integrity:

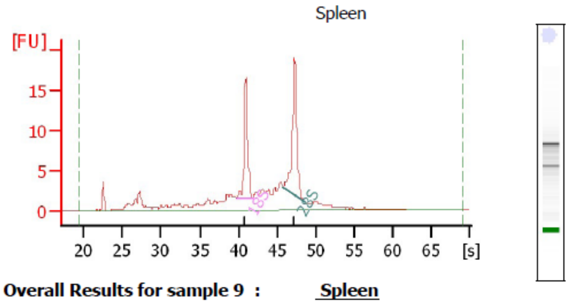

Overall Results for sample 9 : **Spleen**

RNA Area:	123.0
RNA Concentration:	148 ng/µl
rRNA Ratio [28s / 18s]:	1.4
RNA Integrity Number (RIN):	7.9 (B.02.06)
Result Flagging Color:	
Result Flagging Label:	RIN: 7.90

Fragment table for sample 9 : **Spleen**

Name	Start Time [s]	End Time [s]	Area	% of total Area
18S	39.80	41.85	17.6	14.3
28S	45.80	48.55	24.2	19.7

Quantitation and Purity:

260/280: 2.00

260/230: 2.21

Concentration (ng/µl): 500

Stomach

Integrity:

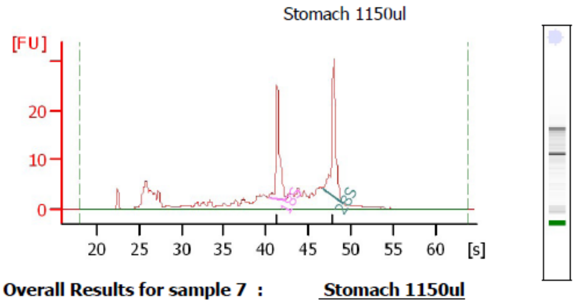

Overall Results for sample 7 : **Stomach 1150ul**

RNA Area:	197.1
RNA Concentration:	180 ng/µl
rRNA Ratio [28s / 18s]:	1.3
RNA Integrity Number (RIN):	7.8 (B.02.06)
Result Flagging Color:	
Result Flagging Label:	RIN: 7.80

Fragment table for sample 7 : **Stomach 1150ul**

Name	Start Time [s]	End Time [s]	Area	% of total Area
18S	40.32	42.41	26.3	13.3
28S	46.67	49.17	33.0	16.7

Quantitation and Purity:

260/280: 2.07

260/230: 1.83

Concentration (ng/µl): 1134.7

Spleen (white pulp)

Integrity:

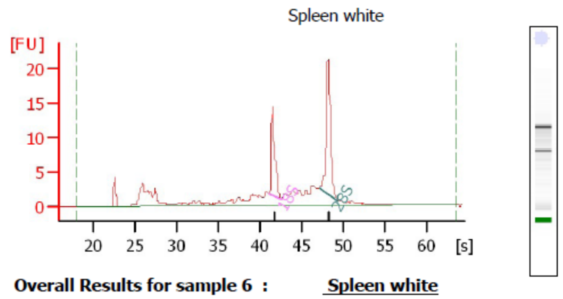

Overall Results for sample 6 : **Spleen white**

RNA Area:	118.3
RNA Concentration:	108 ng/µl
rRNA Ratio [28s / 18s]:	1.5
RNA Integrity Number (RIN):	8.1 (B.02.06)
Result Flagging Color:	
Result Flagging Label:	RIN: 8.10

Fragment table for sample 6 : **Spleen white**

Name	Start Time [s]	End Time [s]	Area	% of total Area
18S	41.13	42.56	15.6	13.2
28S	47.12	49.47	22.9	19.4

Quantitation and Purity:

260/280: 2.02

260/230: 1.83

Concentration (ng/µl): 928.6

Synovial Membrane

Integrity:

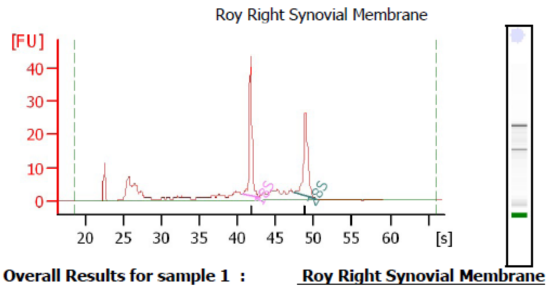

Overall Results for sample 1 : **Roy Right Synovial Membrane**

RNA Area:	188.0
RNA Concentration:	114 ng/µl
rRNA Ratio [28s / 18s]:	0.9
RNA Integrity Number (RIN):	8.2 (B.02.06)
Result Flagging Color:	
Result Flagging Label:	RIN: 8.20

Fragment table for sample 1 : **Roy Right Synovial Membrane**

Name	Start Time [s]	End Time [s]	Area	% of total Area
18S	40.64	42.85	37.2	19.8
28S	47.54	50.36	32.6	17.3

Quantitation and Purity:

260/280: 2.04

260/230: 2.24

Concentration (ng/µl): 966

Tendon (super digital flexor, foal)

Integrity:

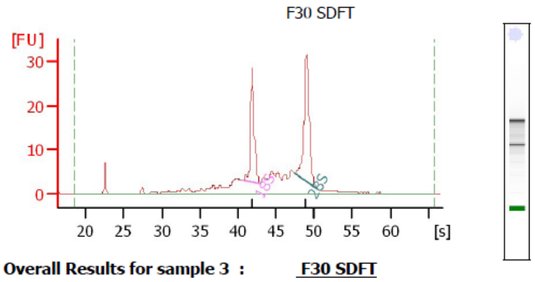

Overall Results for sample 3 : F30 SDFT

RNA Area:	208.8
RNA Concentration:	161 ng/µl
rRNA Ratio [28s / 18s]:	1.3
RNA Integrity Number (RIN):	8.2 (B.02.06)
Result Flagging Color:	
Result Flagging Label:	RIN: 8.20

Fragment table for sample 3 : F30 SDFT

Name	Start Time [s]	End Time [s]	Area	% of total Area
18S	40.61	42.85	35.4	16.9
28S	47.48	50.48	47.2	22.6

Quantitation and Purity:

260/280: 2.01

260/230: 2.12

Concentration (ng/µl): 497

Testes

Integrity:

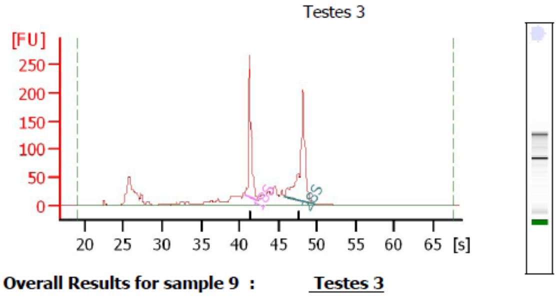

Overall Results for sample 9 : Testes 3

RNA Area:	1,387.9
RNA Concentration:	840 ng/µl
rRNA Ratio [28s / 18s]:	1.4
RNA Integrity Number (RIN):	8.5 (B.02.06)
Result Flagging Color:	
Result Flagging Label:	RIN: 8.50

Fragment table for sample 9 : Testes 3

Name	Start Time [s]	End Time [s]	Area	% of total Area
18S	40.64	42.40	237.1	17.1
28S	45.83	49.66	337.8	24.3

Quantitation and Purity:

260/280: 2.05

260/230: 1.91

Concentration (ng/µl): 1434

Thymus

Integrity:

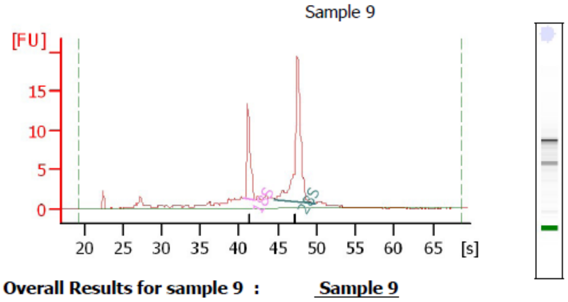

Overall Results for sample 9 : Sample 9

RNA Area:	97.2
RNA Concentration:	140 ng/µl
rRNA Ratio [28s / 18s]:	2.2
RNA Integrity Number (RIN):	8.8 (B.02.06)
Result Flagging Color:	
Result Flagging Label:	RIN: 8.80

Fragment table for sample 9 : Sample 9

Name	Start Time [s]	End Time [s]	Area	% of total Area
18S	40.67	42.11	15.3	15.8
28S	44.60	49.73	34.0	35.0

Quantitation and Purity:

260/280: 2.01

260/230: 2.00

Concentration (ng/µl): 533.83

Vena Cava

Integrity:

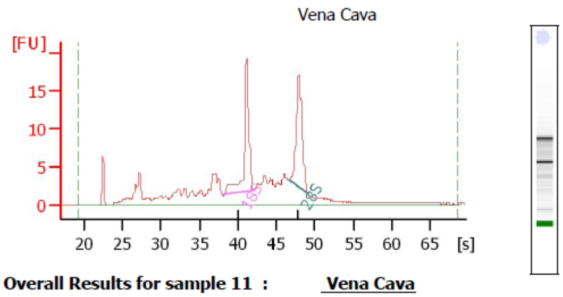

Overall Results for sample 11 : Vena Cava

RNA Area:	193.0
RNA Concentration:	113 ng/µl
rRNA Ratio [28s / 18s]:	0.9
RNA Integrity Number (RIN):	7.6 (B.02.06)
Result Flagging Color:	
Result Flagging Label:	RIN: 7.60

Fragment table for sample 11 : Vena Cava

Name	Start Time [s]	End Time [s]	Area	% of total Area
18S	38.10	42.12	30.9	16.0
28S	46.69	49.22	26.8	13.9

Quantitation and Purity:

260/280: 2.04

260/230: 2.19

Concentration (ng/µl): 500
