# Supplementary figures and images for: Annotation of the Protein Coding Regions of the Equine Genome
Source: PLoS One. 2015 Jun 24;10(6):e0124375. doi: 10.1371/journal.pone.0124375 (PMC4481266; doi:10.1371/journal.pone.0124375)

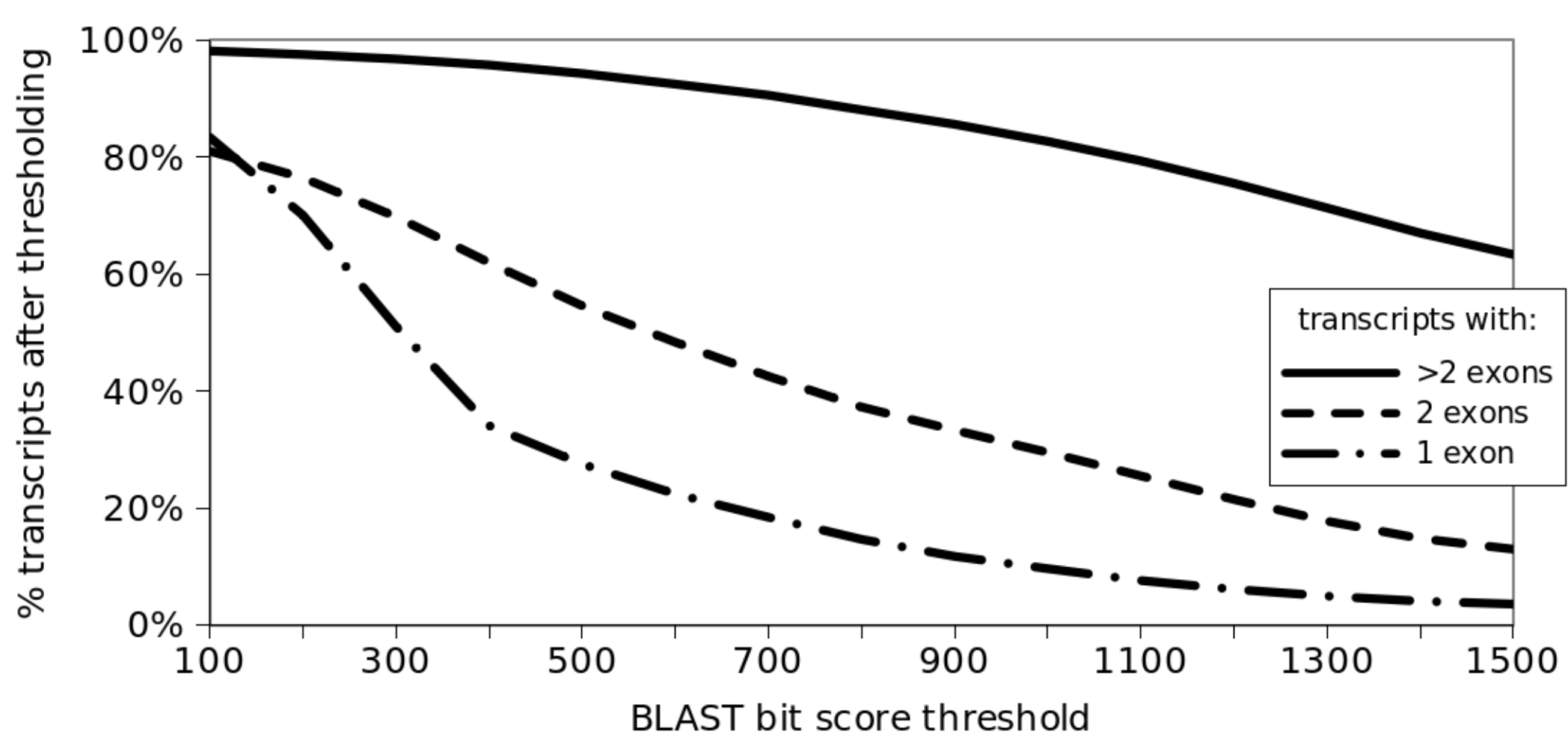

Supplement: S1 Fig — (PDF) [file pone.0124375.s003.pdf]
